# Interpreting Quantifier Scope Ambiguity: Evidence of Heuristic First, Algorithmic Second Processing

**DOI:** 10.1371/journal.pone.0081461

**Published:** 2013-11-20

**Authors:** Veena D. Dwivedi

**Affiliations:** Department of Applied Linguistics and the Centre for Neuroscience, Brock University, Niagara Region, St. Catharines, Ontario, Canada; University of Leicester, United Kingdom

## Abstract

The present work suggests that sentence processing requires both heuristic and algorithmic processing streams, where the heuristic processing strategy precedes the algorithmic phase. This conclusion is based on three self-paced reading experiments in which the processing of two-sentence discourses was investigated, where context sentences exhibited quantifier scope ambiguity. Experiment 1 demonstrates that such sentences are processed in a shallow manner. Experiment 2 uses the same stimuli as Experiment 1 but adds questions to ensure deeper processing. Results indicate that reading times are consistent with a lexical-pragmatic interpretation of number associated with context sentences, but responses to questions are consistent with the algorithmic computation of quantifier scope. Experiment 3 shows the same pattern of results as Experiment 2, despite using stimuli with different lexical-pragmatic biases. These effects suggest that language processing can be superficial, and that deeper processing, which is sensitive to structure, only occurs if required. Implications for recent studies of quantifier scope ambiguity are discussed.

## Introduction

Many levels of information have to be integrated during the complex yet effortless task of language comprehension. For example, word level meaning must be integrated into a phrase and sentence, the structure of which must be consistent with previous context. All this occurs despite the inherent ambiguity present in language at each of these levels. The question addressed in the present work is: what are the underlying mechanisms that allow for language comprehension to occur so efficiently? Furthermore, how are such mechanisms co-ordinated?

The interpretation of sentences displaying semantic ambiguity is presently examined. Sentences of the form *Every kid climbed a tree*, which display quantifier scope ambiguity, have two possible interpretations. Either it is the case that several trees were climbed (on a reading where, for every kid, there is a tree, such that the kid climbed it) or just one tree was climbed (on an analysis where, there is a tree, such that every kid climbed it). The former reading is called the surface scope reading, and the latter is called the inverse scope interpretation. Quantifier scope ambiguous sentences are interpreted according to the order in which the quantifiers *every* and *a* (known in logic as the universal and existential quantifiers, respectively) are interpreted. Of note, the two different readings are characterized according to how many trees are plausibly inferred in the situation; either there are several or just one. The surface scope reading (which is consistent with the surface linear order of the quantifiers in the sentence) is associated with the inference of several trees; henceforth called the plural interpretation. In contrast, the inverse scope reading (where the order of interpretation of the quantifiers is the inverse of linear order) is associated with the single interpretation of *tree*; henceforth called the singular interpretation. Linguists and philosophers have ascribed the following notation as a way of representing the intuitive readings as noted above:

(1) a. (∀x) (x is a kid (∃y) (y is a tree & x climbed y))
[read as: “For every kid x, there is a tree y, such that x climbed y”]
b. (∃y) (y is a tree & (∀x) (x is a kid x climbed y))
[read as: “There is a tree y, such that for every kid, x, x climbed y”]

On one common syntactic account, quantifier scope order preference is represented via quantifier raising, a movement operation assumed to occur at the level of Logical Form [1], [2]. If language processing were assumed to operate on a ‘syntax first’ approach, the ‘deep’ algorithmic computation of scope (as modeled in (1), or some version thereof) would be expected as the driving principle for interpreting quantifier scope ambiguous sentences. ‘Syntax first’ approaches assume that sentences are immediately analyzed according to their syntactic structure, without input from other sources of information, such as lexical-pragmatic knowledge of real-world events, prosodic constraints, visual context, etc. [3–6]. The sentence structure interpretation that is preferred is the one that requires the least amount of structure to build (as in the Minimal Attachment hypothesis of Frazier & Fodor [3]). In the case of quantifier scope ambiguous sentences, an analogous Minimal Structure Hypothesis (as proposed in Dwivedi [7]) would apply at the level of Logical Form during interpretation. In that case, the surface scope interpretation should be preferred (see Text S1 in File S1). Although this mechanism of interpretation has often been called “syntactic,” the more general term *algorithmic computation* will be used here given that we have argued elsewhere that semantic computation is not independent of grammatical considerations [7–9]. Thus, algorithmic computation refers to mechanisms that are sensitive to structural properties of a sentence.

The surface scope preference was revealed in study by Kurtzman & MacDonald [10]. In an end-of-sentence on-line acceptability task, they showed that participants preferred plural continuation sentences such as (2b), after reading quantifier scope ambiguous sentences such as (2a), rather than singular continuations as in (2c).

(2) a. Every kid climbed a tree.
b. The trees were in the park.
c. The tree was in the park.

Thus, participants picked the plural continuation sentence as the better fit with the ambiguous context sentence at about 77% of the time. This result has been replicated in an off-line norming pre-test, reported in an Event Related Potential (ERP) study of quantifier scope ambiguity by Dwivedi, Phillps, Einagel and Baum [11], where the plural continuation was preferred at rates of about 74%. In addition, Raffray and Pickering [12], in a picture priming study, showed that overall, the plural interpretation was the preferred interpretation at about 75% of the time (although this result was not the focus of their study). Thus, that surface scope (consistent with a plural continuation) is preferred for sentences of the form *Every kid climbed a tree* is indeed a robust empirical finding.

That being said, a preference for surface scope interpretation in general has not been fully replicated in several other studies [13–15]. One potential reason why findings have been equivocal is that the above-mentioned studies examined several linguistic factors simultaneously—e.g., type of verb phrase, type of verb, type of quantifier, order of quantifiers. In addition, some of the studies also lacked an unambiguous control condition.

In Dwivedi et al. [11], an ERP language study following up on the results of Kurtzman and MacDonald [10], care was taken such that only ***one*** surface order with active verbs was used; thus *Every N*_*1*_ *Verbed an N*_*2*_, was used for critical stimuli. In addition, only one type of verb phrase (direct object followed by adjunct, described below), and one type and order of quantifiers (*every* followed by *a*) was used. This ERP study examined responses to plural and singular continuation sentences as in (2b,c) which followed quantifier ambiguous context sentences such as (2a). Context sentences were presented in their entirety; participants then pressed a button and after an interstimulus interval of 600 ms, words for the continuation sentence were presented in the centre of the screen at a stimulus onset asychrony (SOA) of 600ms. ERP responses to continuation sentences occurring after quantifier scope ambiguous contexts were compared to those that occurred after unambiguous control contexts, which were *Every kid climbed a different tree* (unambiguous plural) and *Every kid climbed the same tree* (unambiguous singular). Results indicated there was no neurophysiological evidence for a preference of the plural continuation. Instead, plural and singular continuation sentences, following ambiguous context sentences, patterned together (see Text S2 in File S1). These exhibited a late sustained negative-going ERP component 900 ms after the presentation of the noun *tree(s*) in continuation sentences (2b,c) and lasting throughout the presentation of the auxiliary verb *was/were* (for details, see Dwivedi et al. [11]). This slow negative shift (cf. [16], [17]) was interpreted as a reflection of the difficult task of interpreting the previous quantifier scope ambiguous context, which was not fully interpreted after it was presented, and integrating the continuation sentence with such an interpretation. As such, the central claim of Dwivedi et al. [11] was that, at least at very early stages of linguistic analysis, the parser/brain leaves quantifier scope ambiguous sentences as only partially processed, and disambiguation is delayed until further information arrives in the signal.

Thus, the above mentioned study, with its carefully controlled design, did not replicate the preference for the plural continuation sentences. Another possible reason why results have been difficult to replicate could be due to a factor that has yet to be examined in the literature on quantifier scope ambiguity, which is the role of number in event comprehension.

Whereas previous works examined the differing contribution of theta roles and animacy with respect to participants in events [18–24], here the claim is that event representations could have biases regarding the number of participants. Evidence of different biases with respect to number can be found in a follow-up items analysis of the off-line norming study reported in Dwivedi et al. [11]. Briefly, whereas the off-line preference for the plural continuation (evidence of surface scope interpretation) was found 74% of the time, a by-items analysis revealed that not all quantifier scope ambiguous sentences patterned in the same way. That is, the plural continuation was judged as the preferred continuation for a subset of stimuli (such as *Every kid climbed a tree*) at rates close to 100%. Another subset of stimuli (such as *Every jeweller appraised a diamond*) was judged as plural at rates closer to 50%. These differing rates of acceptability underline the importance of the lexical-pragmatic contributions to meaning. In other words, since these sentences all had exactly the same unambiguous syntactic structure, structural considerations cannot explain why the sentence *Every kid climbed a tree* exhibited a strong bias between plural vs. singular continuations, whereas *Every jeweller appraised a diamond* did not. Ostensibly, the explanation would lie in the differing contributions of the particular N_1_VN_2_ lexical items, and the likely events that accompany the interpretation of *kid* ** *climb* ** *tree* vs. *jeweller* ** *appraise* ** *diamond*. Given that experiments examining quantifier scope ambiguity have not controlled for different number biases associated with different events, yet have relied on number interpretation for the disambiguation of quantifier scope ambiguous sentences (as in 2b,c), it could be the case that the lack of replication reported in the literature on quantifier scope ambiguity processing is the result of mixing stimuli with different biases within and across experiments (where the term ‘bias’ refers to the empirically observed interpretation preferences by participants, rather than a tendency as predicted by an algorithmic parsing strategy, such as Minimal Attachment). In the present work, self-paced reading methodology is used to follow up on the previous ERP language experiment of Dwivedi et al. [11], and importantly, stimuli are separated with respect to (the above mentioned empirically observed) number bias as a way of investigating the role of lexical-pragmatic heuristics in quantifier scope interpretation.

A well-known example of a heuristic processing strategy is the N_1_VN_2_ strategy [25], [26], which consists of recognizing that, for the most part, English sentences are structured such that the first Noun (N_1_) is the subject of a sentence and the one following the Verb (N_2_) is the object. Another example of a heuristic processing strategy is using the lexical-pragmatic association of words for interpretation. Note that, in the literature, this heuristic strategy has alternatively been called the *semantics processing stream, lexical association, semantic association*, and more recently, *semantic attraction* (for recent examples, see 27], [28], [21). The idea is that, in the absence of any grammatical/structural information, representations regarding events can be computed by simply recognizing the lexical-pragmatic association of words alone, e.g., *boy* ** *eat* ** *apple* will always be understood as an apple-eating event by a boy. This sort of event interpretation relies on experience with the real-world; it is independent of grammatical computation. Recent ERP language work by Chwilla and Kolk [29] showed that when the final word in a triplet is unexpected (e.g., *vacation* ** *trial* ** *dismissal* vs. *director* ** *bribe* ** *dismissal*) an N400 component is elicited, where this waveform is a marker of lexical-pragmatic anomaly [30], [31]. Thus, even without grammatical cues, simple word triplets can result in an event interpretation (also known as a script or schema, [32], [33]). Consequently, the language processor can posit an event interpretation by quickly scanning incoming linguistic material via simple word recognition, without consulting detailed syntactic and semantic rules of computation. This would result in a ‘good enough’ representation, using ‘quick and dirty’ heuristic processing strategies only [25–27], [34–40].

In sum, two possible routes for sentence interpretation have been proposed for language processing. For our purposes, quantifier scope ambiguous sentences could be interpreted by either a (deep) algorithmic computation, modeled on (1), sensitive to structural analysis (see Text S3 in File S1), or a (shallow) ‘quick and dirty’ lexical-pragmatic heuristic processing strategy, which is independent of structural considerations.

Next, it is an open question as to how these two independent processing streams interact. That is, does one processing stream apply before the other, or do these streams work in parallel (cf. [41], [21]), such that they continuously affect each other? The timing of the application of these streams is up for debate. In contrast to the ‘syntax first’ approach discussed above, recent ERP language work suggests that these streams work in parallel, where the stream with the strongest cue determines whether a P600 effect (evidence of structural processing) vs. an N400 effect (evidence of lexical-pragmatic bias) occurs. However, although ERP methods are renowned for the moment-by-moment timing information that can be gleaned, the standard rate of presentation of words in most ERP language experiments is quite slow (between 300-600 ms per word). As a result, even if the quick and dirty lexical-pragmatic heuristic were to apply in say, the first 300 ms of perception, the slow rate of word presentation would allow for the second phase of algorithmic computation to begin to apply. This would end up looking like parallel and interactive processing as a result. The key is to use a method that does not constrain rate of presentation, so that language processing can apply more “naturally” (modulo a laboratory setting). Furthermore if heuristic and algorithmic phases are ordered sequentially, then measurements at both early and late points of processing are necessary as a way of capturing these independent streams of processing. 

Thus, in the present work, the shallow processing claim regarding quantifier scope ambiguous sentences in Dwivedi et al. [11] is extended and clarified, especially with respect to issues regarding the independent processing streams involving lexical-pragmatic heuristic strategies vs. algorithmic computation and their respective timing. It could be the case that the daunting aspect of quantifier scope interpretation (see 1) results in a processing strategy that *only* uses a quick and dirty lexical-pragmatic heuristic when dealing with quantifier scope ambiguous sentences, (see Text S4 in File S1). In other words, the deep algorithmic computation could occur later than the heuristic strategy (cf. 26) and perhaps *only* if demanded by a task. Thus, it could be the case that participants did not resolve the meaning of quantifier scope ambiguous sentences in Dwivedi et al. [11] because they were never asked to do so (see Text S5 in File S1); only superficial content questions were used in filler trials. This is consistent with the claim made in Swets et al. [38], where it was shown that readers are strategic in terms of how they interpret sentences; for some constructions, readers process deeply only when required to do so. Thus, the experiments below independently investigate the role of task modulation and lexical-pragmatic biases in sentences exhibiting quantifier scope ambiguity. In doing so, these experiments will build on the ERP findings of Dwivedi et al. [11] and clarify how sentences are interpreted and integrated into semantically ambiguous contexts. Furthermore, the hypothesis that language processing does not invoke deep algorithmic processing (unless required to do so) will be investigated. The stimuli under investigation are carefully modeled after previously published works [10–12], using stimuli biases from Dwivedi et al. [11]. The factors of interest are Context (2 levels: Ambiguous, Unambiguous) and Number (2 levels: Plural, Singular). See Table 1 for samples of experimental stimuli.

**Table 1:** Sample Critical Stimuli.

	**Context**	
**Number (continuation)**	**Ambiguous**	**Unambiguous**
Plural	Every kid climbed a tree. The trees were in the park.	Every kid climbed those trees. The trees were in the park.
Singular	Every kid climbed a tree. The tree was in the park.	Every kid climbed that tree. The tree was in the park.

In the first experiment, sentences that are heavily biased (93-100%) for the plural continuations (consistent with surface scope interpretation) are presented in a self-paced reading study. If heuristic and algorithmic processing streams occur in parallel, then a strong bias in favour of plural continuation sentences should occur (see Text S6 in File S1). Therefore, reading times (RTs) for plural conditions when following ambiguous contexts should not differ from those following unambiguous contexts (since both are congruent with expectations). In contrast, the singular continuation sentence should exhibit longer RTs following ambiguous contexts than those following unambiguous singular control contexts, since the plural interpretation would be expected after ambiguous contexts. Furthermore, given the lateness of the effect found in the previous ERP experiment [11], effects should occur towards the end of the continuation sentence. On the other hand, on a heuristic first model, shallow processing of the context sentence could result in superficial processing of the continuation sentence, such that no real integration occurs. This would result in a lack of a difference between continuation sentences.

In the second experiment, the same heavily biased stimuli are used but are now followed by explicit questions regarding the interpretation of sentences. These questions should modulate the depth of processing so that participants now pay attention, and an effort to integrate continuation sentences should occur. If heuristic and algorithmic processing streams occur in parallel, data should pattern as predicted for Experiment 1; in fact, deeper processing could result in an enhancement of the predicted RT difference expected for singular continuations following ambiguous contexts (vs. unambiguous control singular contexts). Question-response accuracy will also yield information regarding participants’ actual interpretation of sentences. A difference between ambiguous singular vs. unambiguous singular conditions is expected, such that accuracy rates should be lower for the ambiguous singular condition; whereas response accuracy for ambiguous plural should be quite high, and unambiguous plural conditions should reflect accuracy rates close to ceiling. In other words, the pattern of responses for RTs and question-response accuracy should be similar.

 Alternatively, if the heuristic phase precedes the algorithmic phase in terms of timing, then the pattern found for on-line reading would differ from that found for mean question-response accuracy. In this case, the prediction is that the self-paced reading time data, representative of the quick and dirty heuristic phase, would only reflect the lexical-pragmatic biases of the stimuli. This would result in an effect of Number at the continuation sentence, where singular sentences would take longer to read than plural sentences. No effect of ambiguity is expected on this account, since the lexical pragmatic bias in this experiment only concerns Number. However, the question-response accuracy rates should still result in the ambiguous singular condition having the lowest accuracy rate of all conditions, since this task would require algorithmic interpretation. 

Experiment 3 follows up on the findings of Experiment 2; it involves stimuli without a strong lexical-pragmatic bias, such that these sentences are truly ambiguous with respect to quantifier scope interpretation (e.g., *Every jeweller appraised a diamond*). Should heuristic and algorithmic strategies occur in parallel, patterns found in on-line RT data should mirror question-response accuracy. On this view, given that there is no strong lexical-pragmatic cue for interpreting scope ambiguous context sentences in Experiment 3 [28], [41], the algorithmic stream should immediately do the work to disambiguate the meaning of context sentences, resulting in a surface scope preference. As a consequence, the singular continuation following ambiguous contexts should be dispreferred, resulting in longer RTs and lowest accuracy rates in response to questions. Thus, on-line RTs and question-response accuracy should yield similar patterns in both Experiments 2 and 3, such that findings indicate that the ambiguous singular condition is dispreferred, on an account where both heuristic and algorithmic processing streams occur in parallel.

In contrast, if the heuristic phase precedes the algorithmic phase, then a different data pattern is expected to be observed for reading times vs. question-response accuracy responses. Namely, reading time data at continuation sentences should reflect the lexical-pragmatic biases of the stimuli; in Experiment 3, now an effect of Context is predicted. That is, sentences following ambiguous contexts should take longer to read than those embedded in unambiguous contexts, since the former context is more complex. Crucially, no effect of Number is predicted here, since that is not part of the lexical-pragmatic bias in this experiment. Furthermore, whereas patterns associated with RTs are expected to differ from Experiment 2, question-response accuracy rates should not. The algorithmic computation is only sensitive to structural considerations and should be independent of lexical-pragmatic biases. In other words, RT results in Experiment 3 should differ from those of Experiment 2 (reflecting the different lexical-pragmatic bias), but question-response accuracy rates should not (reflecting the same algorithmic computation). See Table 2 for an overview of the experiments in the present work.

**Table 2:** Overview of Experiments in the Present Work.

**Experiment**	**Lexical-Pragmatic bias?**	**Task demands?**
**1**	yes	no
**2**	yes	yes
**3**	no	yes

## Experiment 1

### Materials and Methods

#### Ethics statement

This study received ethics approval from the Brock University Social Science Research Ethics Board (SREB) prior to the commencement of the experiment (REB 07-293). Written, informed consent was received from all participants prior to their participation in the experiment.

#### Participants

Eighty right-handed native speakers of English (59 female, mean age 22 years, range 18 to 34 years) were recruited at Brock University and were either paid $10 each to participate in the experiment or were given partial course credit (if applicable). 

#### Materials

Twenty-four experimental stimuli were prepared such that each consisted of 2 sentences: a context sentence (Sentence 1, S1) and a continuation sentence (Sentence 2, S2). See Table 1. The context sentence always began with *Every NP* as a subject, and the direct object was either a Noun Phrase (NP) preceded by an existential quantifier (a) for ambiguous contexts, or a referential determiner (*that/those*) for unambiguous contexts. The use of these determiners would ensure that no scope ambiguity could occur with these conditions [42] (see Text S7 in File S1). The continuation sentences began with a singular or plural subject NP and auxiliary verb (*The tree(s*)* was/were; the melon(s*)* was/were*), followed by either a prepositional phrase (*in the park*) or conjoined adjectives (*soft and juicy*). 

The 24 experimental items were combined with 64 stimuli from an unrelated experiment, and 64 fillers, for a total of 152 items. Four lists were created in order to ensure that the conditions were counterbalanced as per Latin square design. In order to ensure that participants were paying attention to the experiment, the 64 filler items were followed by simple questions pertaining to their superficial content. The questions were forced choice, with two buttons (labeled as “1” and “2”) designated for answer selection. An example stimulus/question pair is shown in (3):

(3) Because of the thunderstorm, Lara had trouble sleeping.
She felt terrible the next day.
Did Lara sleep well? 1) Yes 2) No

Participants pressed the button that corresponded to the answer on the screen. Answers were counterbalanced such that equal numbers of correct answers were displayed on the right and left side of the screen.

**Figure 1:**
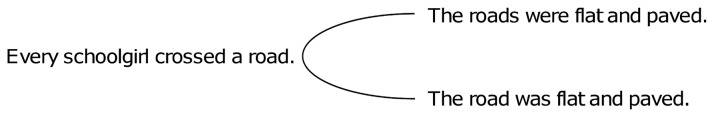
Example of an ambiguous pre-test item in Dwivedi et al. (2010). Participants were instructed to circle the continuation sentence (e.g. *The* *roads* *were* *flat* and *paved* or *The* *road* *was* *flat* and *paved*) that best fit with the first sentence (e.g. *Every schoolgirl crossed a road*).

The 24 items used in the present study were 93-100% plurally biased, i.e., heavily biased for surface scope interpretation (see Critical Stimuli List S1 in File S1 for a list of critical stimuli and biases). These sentences were selected from a previous off-line study reported in Dwivedi et al. [11]. Two semi-randomized lists were created, and 32 subjects (none of whom participated in the present experiment) read ambiguous context sentences as above, and were asked to circle the preferred continuation sentence (see Figure 1). In this off-line task, discourses were presented in a booklet in a pseudo-random order, with the constraint that no more than two of the same type of trial succeeded one another. In each list, 80 ambiguous context sentences were presented, as well as 80 unambiguous ones (40 unambiguous singular and 40 unambiguous plural). Note that plural and singular continuation sentence choices were counterbalanced to appear either on the top or bottom position. In addition, 80 fillers were used from an unrelated experiment. Results were consistent with those of Kurtzman and MacDonald [10], such that the plural continuation sentence *The trees were in the park* was preferred for Ambiguous contexts such as *Every kid climbed a tree* 74% of the time. For the current study, an items analysis was conducted. Results indicated that not all items were biased in the same way, such that the plural preference ranged from 20-100%. Sentences most heavily biased for plural interpretation were used for this (and the following) experiment.

#### Procedure

The on-line task involved self-paced reading, word-by-word, with a moving window display [43] (see Text S8 in File S1). All non-space characters of both the context sentence (S1) and the continuation sentence (S2) were presented on one screen masked by dash symbols (-). S2 always began on a new line on the left margin adhered to by S1, and the same applied for lengthy sentences which occupied more than one line. Participants pressed a button to advance from word to word, such that only one word was visible on the screen at a time. Reading time was recorded as the time between button presses. Before starting the experiment, participants practiced on a short list of items in order to familiarize themselves with task requirements.

E-Prime software was used to present the self-paced reading task. A 19” widescreen Dell LCD monitor was approximately 18-24 inches from the participant, level with the participant’s point of view. The order of sentence presentation was randomized per participant by E-Prime software. Participant responses were recorded via a PSTnet serial response button box. 

### Results

Data were filtered such that any data point that was more than 2 standard deviations away from the mean (within subject, condition, and word position) in either direction was attenuated to the nearest ceiling value. This affected less than 2% of the data.

All statistical analyses concerned reading times recorded per word at S1 and S2. Analyses were also conducted using residual reading times (RRT; see 4], [44). Because the pattern of results obtained for RRT analyses was exactly analogous to the raw RT data, the results are reported with respect to the latter dependent variable only.

The reading time analyses were based on PASW (see Text S9 in File S1) v18 statistical software and the Greenhouse-Geisser [45] non-sphericity correction was employed for effects with more than one degree of freedom in the numerator. Following convention, unadjusted degrees of freedom are reported, along with adjusted *p*-values. Mean square error values reported are those corresponding to the Greenhouse-Geisser correction. All significant main effects, by participants and by items [46], involving the factors of interest (Context and Number) are reported first, followed by the highest order interaction effects involving Context and/or Number. Effect size is reported using partial eta squared, ŋ_p_^2^.

Separate repeated measure ANOVAs were conducted for S1 and S2. 

#### Comprehension question performance

The mean question-response accuracy for participants was at 96%, indicating that participants were indeed paying attention. 

#### Reading times at context sentence (S1)

Given that ambiguous vs. unambiguous context sentences are exactly alike until after the verb, analyses were run at the final word of S1, *tree(s*). This ANOVA was defined by the within-subjects factors of Context (2 levels: ambiguous vs. unambiguous) and Number (2 levels: plural vs. singular). A main effect of Context was found; this was significant by participants but not by items (*F*_*1*_ (1,79) = 12.14, MSE = 7644; *p* = .001, ŋ_p_^2^=.133; *F*_*2*_ (1,23) = 176, MSE = 19954, *p* =.19, ŋ_p_^2^=.071). Reading times to unambiguous contexts took 34 ms longer than ambiguous contexts, i.e., 460ms vs. 426ms. No other effects were significant.

#### Reading times at continuation sentence (S2)

**Figure 2:**
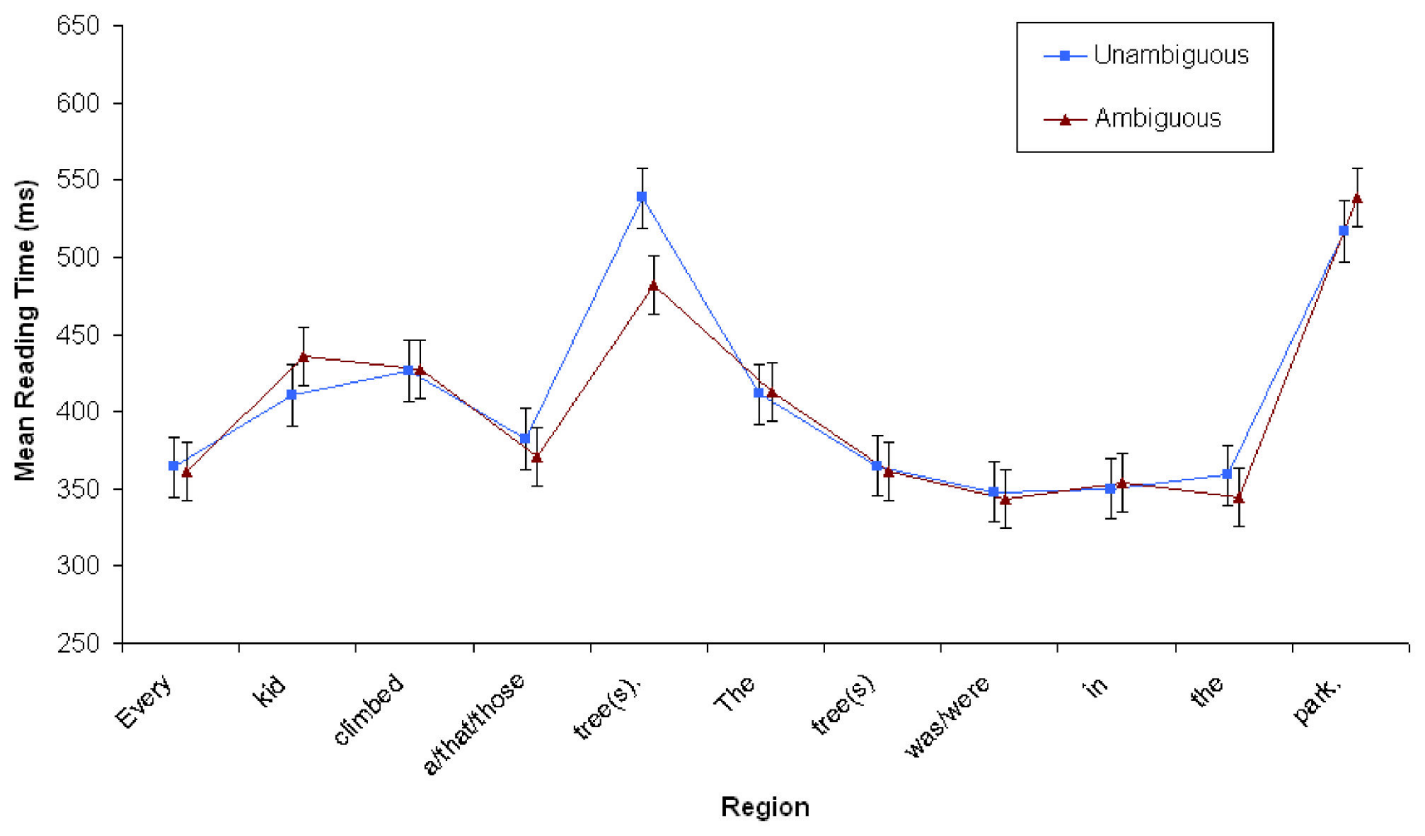
Reading time in milliseconds at both S1 and S2 in Experiment 1 (*N*=80). Points represent mean RTs per word, with vertical lines depicting the standard error of the means.

A repeated measures ANOVA was conducted for the whole sentence at S2. Word positions in the whole sentence were analyzed because at the face of it, it is unclear where effects might occur. On the one hand, one could expect integration and interpretation to occur at the subject noun position *tree(s*), since once that anaphor is perceived, it needs to be linked with the previous discourse [47], [48]. However, given the lateness of the effect noted in previous work, effects could also occur at the end of the sentence. Thus, besides Context and Number, Word Position was a factor in this analysis (6 levels: Det^N^Verb^V1^V2^V3, i.e., *The^tree*(*s*)*^was/were^in^the^park*). As is evident in Figure 2, no significant effects or interactions were found for Context or Number (all *Fs*<2) in any region of the continuation sentence.

### Discussion

The goal of this experiment was to investigate whether empirical differences could be found between plural vs. singular continuation sentences following quantifier scope ambiguous contexts. It was predicted that the strong lexical-pragmatic bias for the plural continuation for quantifier scope ambiguous sentences (in addition to the same preference for algorithmic computation, should it have applied) would result in the ambiguous singular continuation sentences taking more time to read. Nevertheless, in the experiment above, no differences were found in any continuation sentence, despite the heavily biased stimuli. Clearly, the algorithmic work of scope computation did not occur in real-time. However, it is unclear why results did not reflect heuristic processes—why didn’t plural continuation sentences take less time to read than singular sentences? The lack of a difference found for continuation sentences can be explained using claims in Hannon and Daneman [49], which also examined sentence processing in context. That work suggested that once shallow processing is adopted, which is precisely the processing strategy suggested for scope ambiguous sentences (see Dwivedi et al. [11]), then “the comprehension system may assume coherence as a default, as long as there is sufficient global coherence,” ([49],p. 459; [50]). In other words, once the continuation sentence arrives, no real effort is made for integration, since the interpretation at the context sentence is so shallow. This lack of attention results in a lack of difference for reading times at continuation sentences.

The only difference found was between ambiguous and unambiguous context sentences at S1, whereby participants spent more time reading the unambiguous context sentences than scope ambiguous sentences at the final word. Although this was significant by participants and not by items, it is a pattern that will be observed again, and so deserves discussion here.

The difference at the first sentence was not expected. Two possible explanations emerge. First, it could be the case that participants spend less time at ambiguous vs. unambiguous context sentences because they underspecify these sentences. That is, one way of looking at the data would be to claim that quantifier scope ambiguous sentences are read more quickly than unambiguous ones. This proposal would be congruent with the findings of Traxler, Pickering, and Clifton [51] and Swets et al. [38], where ambiguous sentences were shown to have shorter reading times than unambiguous sentences. A faster reading time is consistent with shallow processing of quantifier scope ambiguous sentences.

Another possible explanation does exist, however. It could be the case that participants are spending more time at the unambiguous context because this context uses the referential NPs *that*/*those tree(s*). Given that these sentences were not preceded by any other linguistic material, *that/those tree(s*) does not actually refer to anything. As such, it could be the case that the significant difference in reading time for participants is their ability to notice this discourse pragmatic anomaly. Future experiments are planned where S1 is preceded by a context sentence to further investigate this finding. 

In Experiment 2, we use the same stimuli (biased for plural) as in Experiment 1, but now participants are given the strategic goal of having to answer questions regarding the interpretation of the two-sentence discourse just read. It is hypothesized that the presence of the questions will force participants to pay more attention to the discourse, such that effort to integrate continuation sentences will be made.

## Experiment 2

### Materials and Methods

#### Ethics statement

This study received ethics approval from the Brock University Social Science Research Ethics Board (SREB) prior to the commencement of the experiment (REB 07-293). Written, informed consent was received from all participants prior to their participation in the experiment.

#### Participants

Forty-eight right-handed native speakers of English (39 female, mean age 20.8 years, range 18 to 30 years) were recruited at Brock University and were either paid $10 each to participate in the experiment or were given partial course credit (if applicable).

#### Materials

The 24 biased target materials were the same as those in Experiment 1. These materials were combined with 64 stimuli from an unrelated experiment, and 101 fillers, for a total of 189 items. Target sentences were divided into four lists, ensuring that all factors were counterbalanced in Latin square format. All trials were followed by a question. That is, in addition to all filler trials being followed by questions (unlike Experiment 1, where this was the case for half), all target sentences were now followed by questions that directly queried their interpretation, as in

(4) Every kid climbed a tree.
The trees were in the park.
How many trees were climbed? 1) Several 2) One

All questions were forced choice, with two buttons (labeled as “1” and “2”) designated for answer selection. Participants pressed the button that corresponded to the answer on the screen. Answers were counterbalanced such that an equal number of correct answers were displayed on the right and left hand side of the screen. The target sentences used were exactly the same as in Experiment 1. See Critical Stimuli List S1 in Supporting Information for a complete list of stimuli.

#### Procedure

The procedure in the present experiment was analogous to Experiment 1 (see Text S10 in File S1).

### Results

Data filtering was completed as in Experiment 1; this affected less than 1% of the data. All statistical analyses were conducted as in Experiment 1. 

#### Reading times at context sentence (S1)

Analyses at the final word of S1 again revealed a main effect of Context (*F*_*1*_ (1, 47) = 6.91, MSE = 43931; *p* = 0.012; ŋ_p_^2^=.128; *F*_*2*_ (1, 23) = 22.21, MSE = 7127; *p* < 0.001; ŋ_p_^2^=.491). 

**Figure 3:**
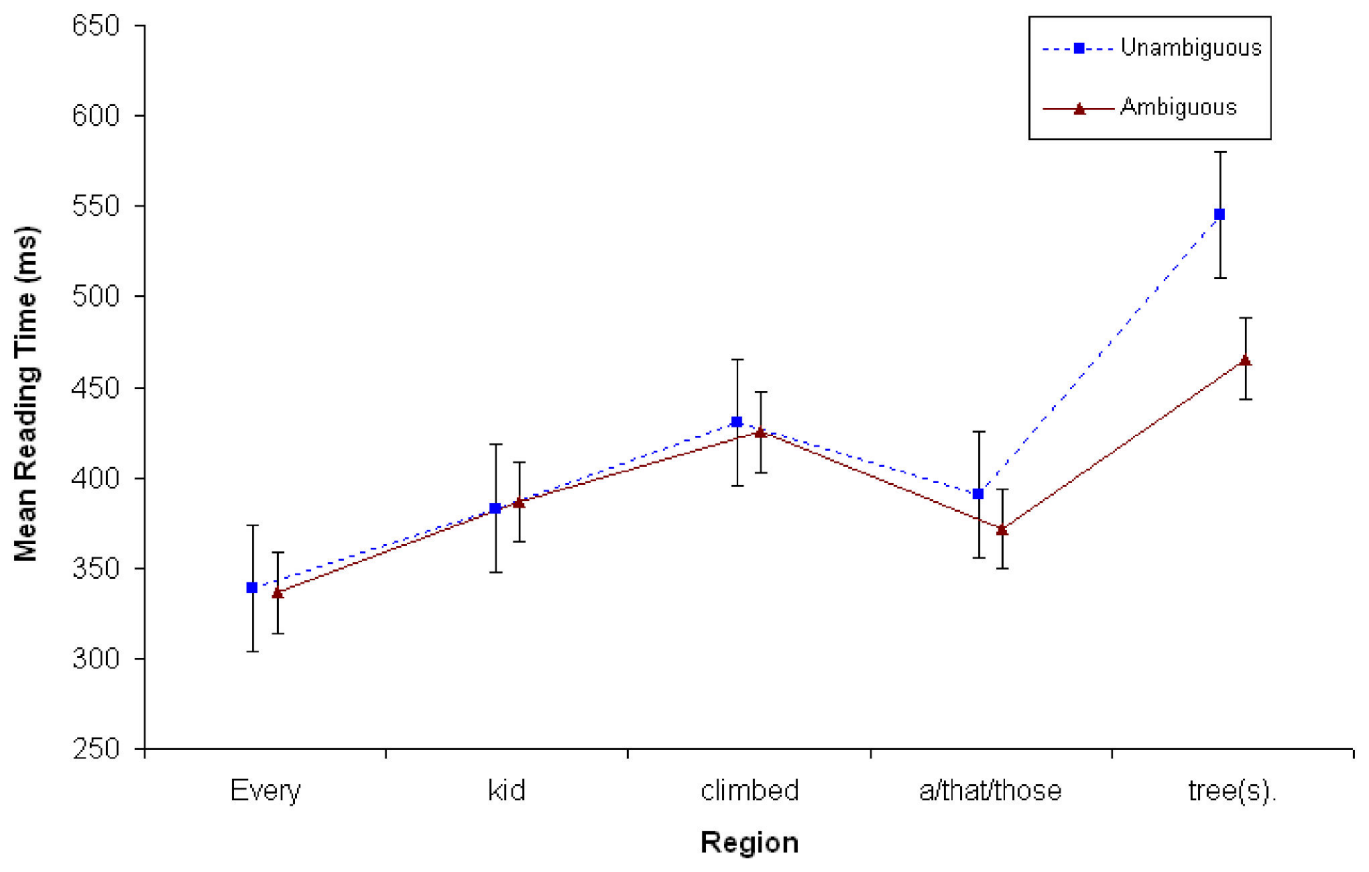
Reading time in milliseconds at S1 in Experiment 2 (*N*=48). Points represent the mean RTs per word; vertical lines depict standard error of the means.

Overall, the unambiguous context sentence took more time to read than the ambiguous contexts (545ms vs. 465ms). See Figure 3.

#### Reading times at continuation sentence (S2)

**Figure 4:**
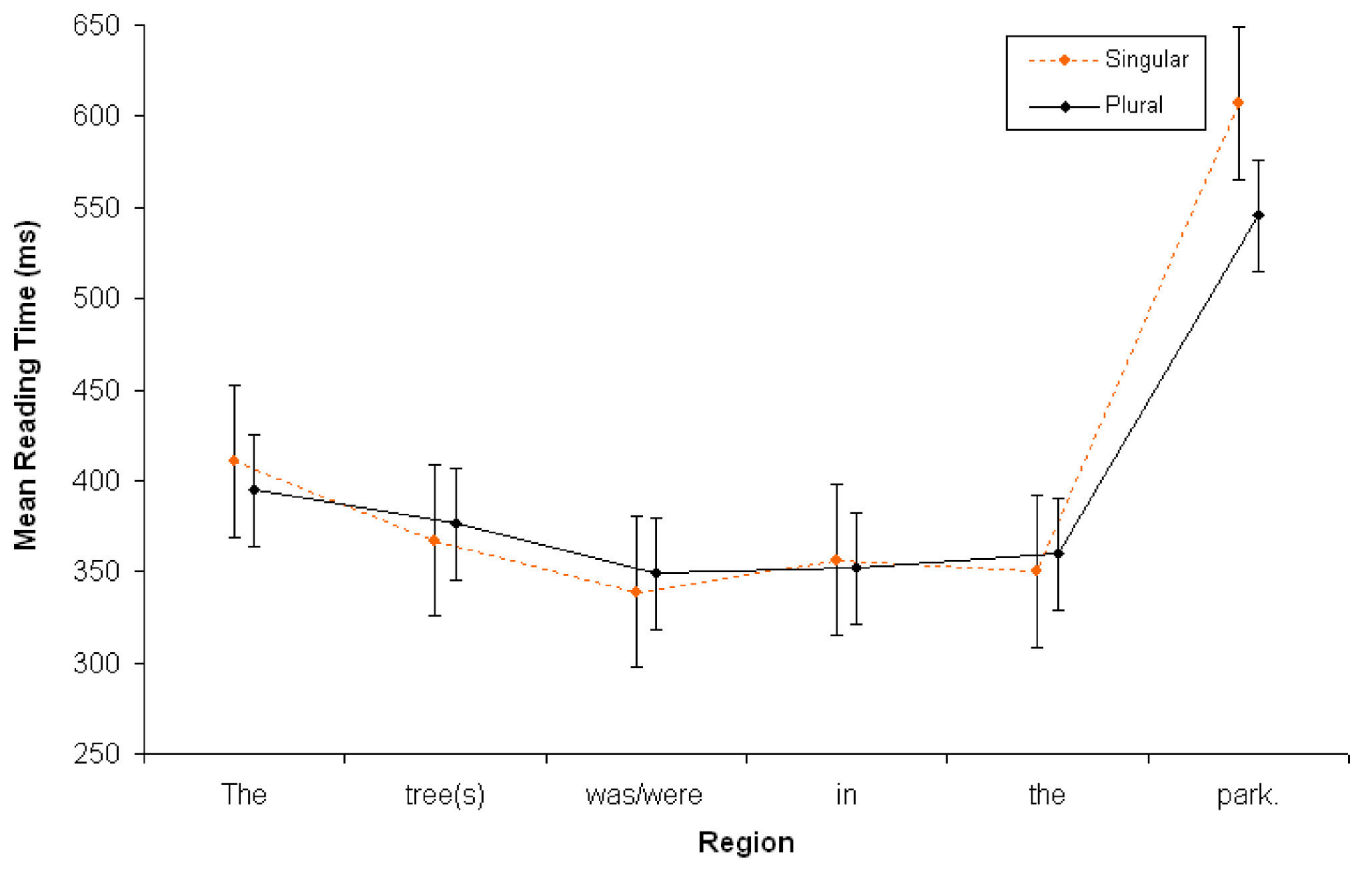
Reading time in milliseconds at S2 in Experiment 2 (*N*=48). Points represent mean RTs per word; vertical lines depict the standard error of the means.

A repeated measures ANOVA was conducted at the second sentence (S2), exactly as in Experiment 1. There were no main effects associated with Context or Number (*Fs*<2). There was a significant interaction between Number x Word Position ((*F*_*1*_ (5, 235) = 6.24, MSE = 58577; *p* < .001; ŋ_p_^2^=.117; *F*_*2*_ (5,115) = 5.84, MSE = 7617; *p* < .001; ŋ_p_^2^=.202). Simple effects analyses revealed that the final word (*park*) took 61ms longer to read in Singular vs. Plural conditions (607ms vs. 545ms), as is evident in Figure 4. Interestingly, there were no significant interactions with Context (*Fs*<2).

#### Question answer results

**Figure 5:**
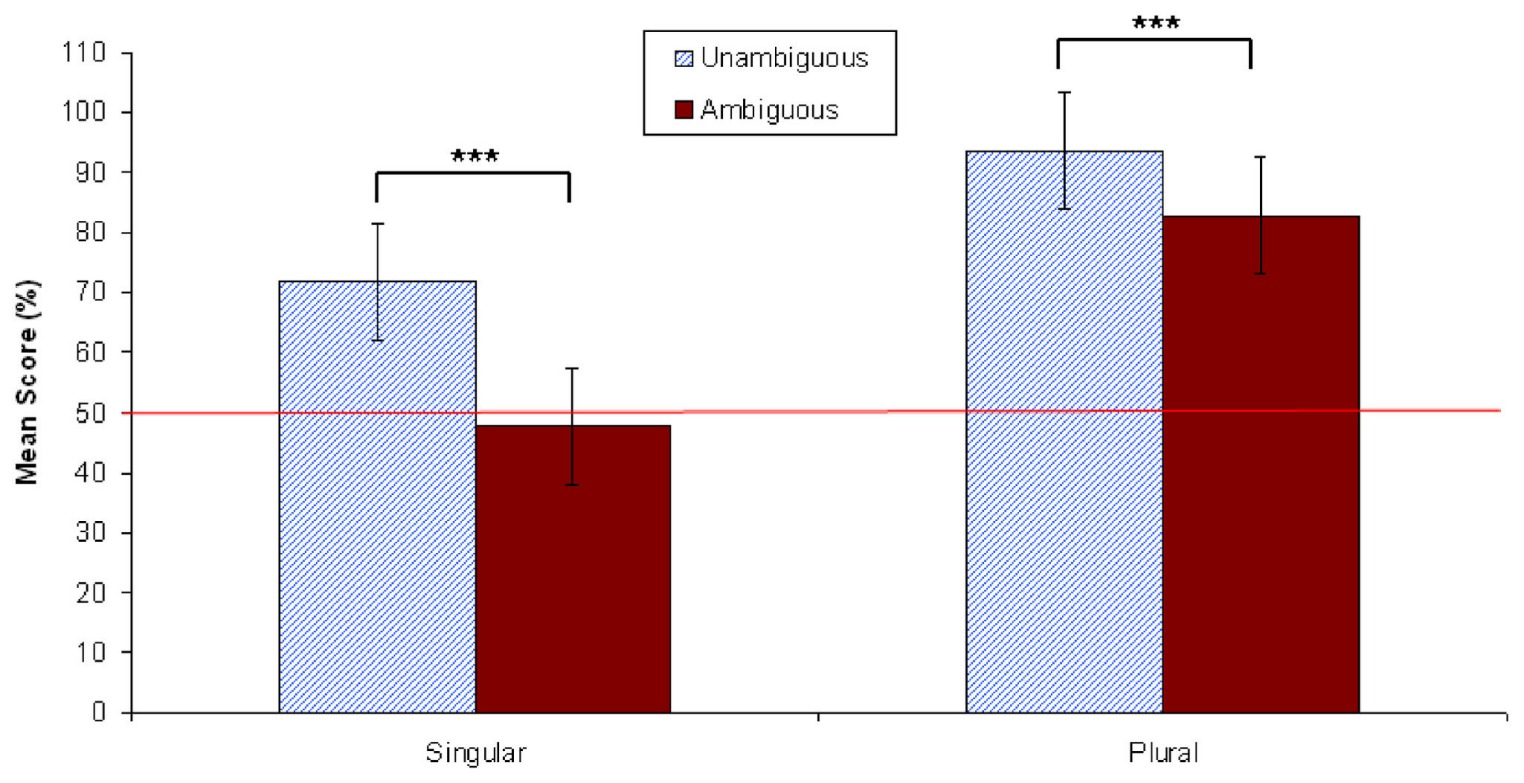
Mean comprehension question accuracy in Experiment 2 (*N*=48). Vertical lines depict standard error of the means, and the horizontal line indicates an accuracy score of 50% (chance). The difference between accuracy for unambiguous and ambiguous singular stimuli was significant at *p* < .001. The difference between accuracy for unambiguous and ambiguous plural stimuli was also significant at *p* < .001. ***p < .001.

**Figure 6:**
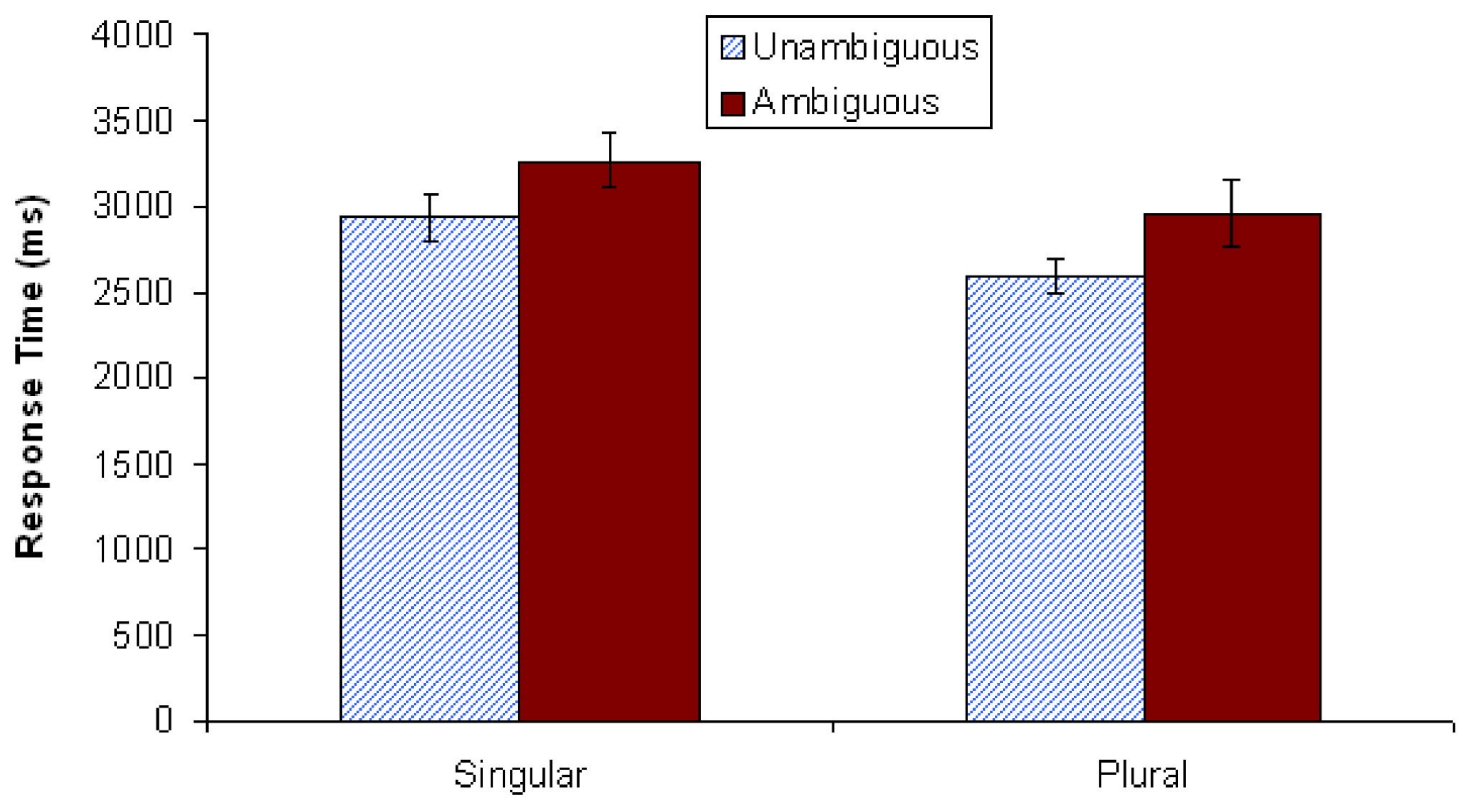
Mean comprehension question-response time in Experiment 2 (*N*=48). Vertical lines depict standard error of the means.

As is shown in Figure 5, participants performed at levels nearly equivalent to chance (47%) when responding to the ambiguous singular condition. That is, when answering *How many trees were climbed*? after having just read a singular continuation sentence embedded in an ambiguous context, participants did extremely poorly. In contrast, the mean accuracy level for the ambiguous plural condition was 83%. Both of these accuracy rates differed significantly from their unambiguous controls, as revealed in paired-samples t-tests (see Figure 5). Note that there is a clear bias towards accuracy in the plural conditions vs. the singular conditions; accuracy for the unambiguous singular condition was at 71% vs. 94% for the unambiguous plural. Furthermore, the lower accuracy rates for the singular conditions cannot be explained via a speed-accuracy trade off. Figure 6 shows that singular conditions took more time to respond to than plural conditions (see Text S11 in File S1).

### Discussion

To summarize the results above, in Experiment 2, results at S1 were similar to those of Experiment 1. That is, a difference at the end of unambiguous vs. ambiguous context sentences was found again, such that the former took longer to read. Furthermore, the addition of questions in this experiment resulted in a reading time difference at the end of S2 for plural vs. singular sentences. Finally, participants’ response accuracy rates were at or slightly below chance for the ambiguous singular condition. Implications of these results are discussed below.

#### Context sentence (S1)

The fact that the final region for scope ambiguous context sentences (*a tree*) took less time to read than the unambiguous conditions (*that/those trees*) in the present experiment and in Experiment 1 discounts the hypothesis that participants took less time at ambiguous contexts because they were not paying attention to these due to underspecification. In the present experiment, task demands required that participants pay more attention to both context and continuation sentences. Under an analysis employed by Swets et al. [38], one might predict that the S1 difference would be mitigated in Experiment 2 because participants tend to spend more time attending to stimuli where they know they will be asked a question. However, instead the pattern of results looks just like Experiment 1. Thus, the difference found at S1 seems to be due to the discourse anomaly associated with using *that* and *those* in unambiguous control contexts. While this finding is of interest in its own right, it is orthogonal to the question at hand.

#### Continuation sentence (S2) and question-response accuracy rates

Unlike the findings of Experiment 1 and Dwivedi et al. [11], a difference between singular and plural continuation sentences was found in Experiment 2. Singular continuation sentences took longer to read at the final word, as compared to plural continuation sentences. There was no effect of quantifier scope ambiguity. Thus, the reading time pattern of continuation sentences mirrored the lexical-pragmatic bias of the context sentence. That is, note that context sentences were heavily biased for the plural interpretation (as per previous off-line results). However, question-response accuracy patterns were markedly different. The ambiguous singular condition was responded to at equal or slightly below chance levels. Given that participants had just read the singular continuation sentence *The tree was in the park*, it is striking that college-aged students should have performed so poorly when interpreting this question. This pattern of results is consistent with a heuristic first, algorithmic second model of language processing. The hypothesis is that by the end of the continuation sentence, only lexical-pragmatic heuristics have applied. That is, the algorithmic parsing strategy does not apply at all during on-line reading. However, once the question task arrives, the algorithmic computation becomes necessary. The considerably low accuracy rate for this condition reflects how the inverse scope interpretation is highly dispreferred, given an algorithmic parsing strategy such as Minimal Attachment. Thus, the pattern of results for Experiment 2 is consistent with a model of language comprehension where a ‘quick and dirty’ lexical-pragmatic phase first attends to linguistic material during on-line reading, followed by a deeper algorithmic parsing phase, which only applies because it was required by the question task. 

Experiment 3 further examines this claim by testing stimuli with a different lexical-pragmatic bias for the plural continuation but with exactly the same unambiguous syntactic structure. If the hypothesis regarding the timing of the application of these independent processing streams is correct, then like Expeiment 2, results for Experiment 3 should show a different pattern for on-line RTs as compared to question-response accuracy data. 

## Experiment 3

### Materials and Methods

#### Ethics statement

This study received ethics approval from the Brock University Social Science Research Ethics Board (SREB) prior to the commencement of the experiment (REB 07-293). Written, informed consent was received from all participants prior to their participation in the experiment.

#### Participants

Forty right-handed native speakers of English (32 female, mean age 20.2 years, range 18 to 25 years) were recruited at Brock University and were either paid $10 each to participate in the experiment or were given partial course credit (if applicable).

#### Materials

The structure of the materials was comparable to Experiments 1 and 2. The 24 ambiguous context sentences were selected from a previous off-line study reported in Dwivedi et al. [11], as was done for Experiments 1 and 2. The only difference here in Experiment 3 was that the 24 items used in the present study were 44-67% plurally biased, i.e., unbiased (see Critical Stimuli List S2 in File S1 for a complete list of critical stimuli). A sample is shown in Table 3.

**Table 3:** Sample Critical Stimuli for Experiment 3 (unbiased).

	**Context**	
**Number (continuation)**	**Ambiguous**	**Unambiguous**
Plural	Every jeweller appraised a diamond. The diamonds were clear and flawless.	Every jeweller appraised those diamonds. The diamonds were clear and flawless.
Singular	Every jeweller appraised a diamond. The diamond was clear and flawless.	Every jeweller appraised that diamond. The diamond was clear and flawless.

#### Procedure

The procedure in the present experiment was identical to Experiment 2.

### Results

Data were filtered using the same procedure as in Experiments 1 and 2; this affected less than 2% of the data.

All statistical analyses reported concern raw reading times recorded per word at the context sentence (S1) and continuation sentence (S2; note that residual reading time analyses yielded parallel results).

Separate repeated measures mixed ANOVAs were conducted for S1 and S2.

#### Reading times at context sentence (S1)

**Figure 7:**
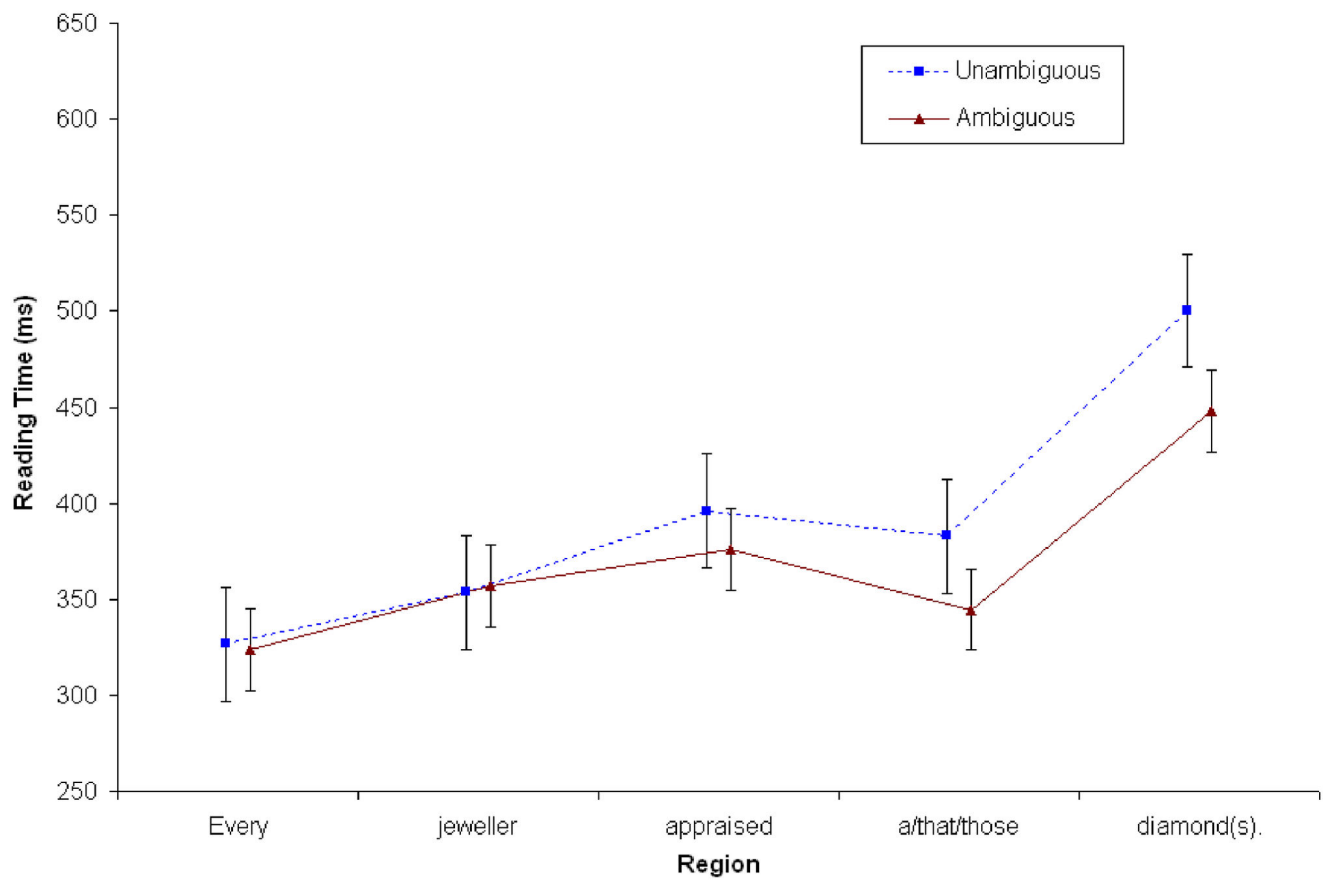
Reading time in milliseconds at S1 in Experiment 3 (*N*=40). Points represent the mean RTs per word; vertical lines depict standard error of the means.

There was a main effect of Context (*F*_*1*_ (1, 39) = 5.88, MSE = 20587; *p* = 0.023; ŋ_p_^2^=.125; *F*_*2*_ (1, 23) = 3.05, MSE = 21617; *p* = 0.09; ŋ_p_^2^=.117). In addition, there was a Context x Number interaction (*F*_*1*_ (1, 39) = 8.73, MSE = 18832; *p* = 0.005; ŋ_p_^2^=.183; *F*_*2*_ (1,23) = 6.77, MSE = 14939; *p* =.016; ŋ_p_^2^=.227). See Figure 7.

Overall, the unambiguous context took more time to read than the ambiguous contexts (439ms vs. 394ms, *p* = .02), much like what was seen in Experiments 1 and 2. However, in Experiment 3 this result is driven by the Context x Number interaction, where the unambiguous singular condition (*that diamond*) took 88ms more time to read than *a diamond* (*p* = .001) and 59 ms more time to read than *those diamonds* (*p* = .01). This effect is what drives the difference to occur earlier (see Figure 7) such that the significant differences now occur at the determiner position *that*.

#### Reading times at continuation sentence (S2)

**Figure 8:**
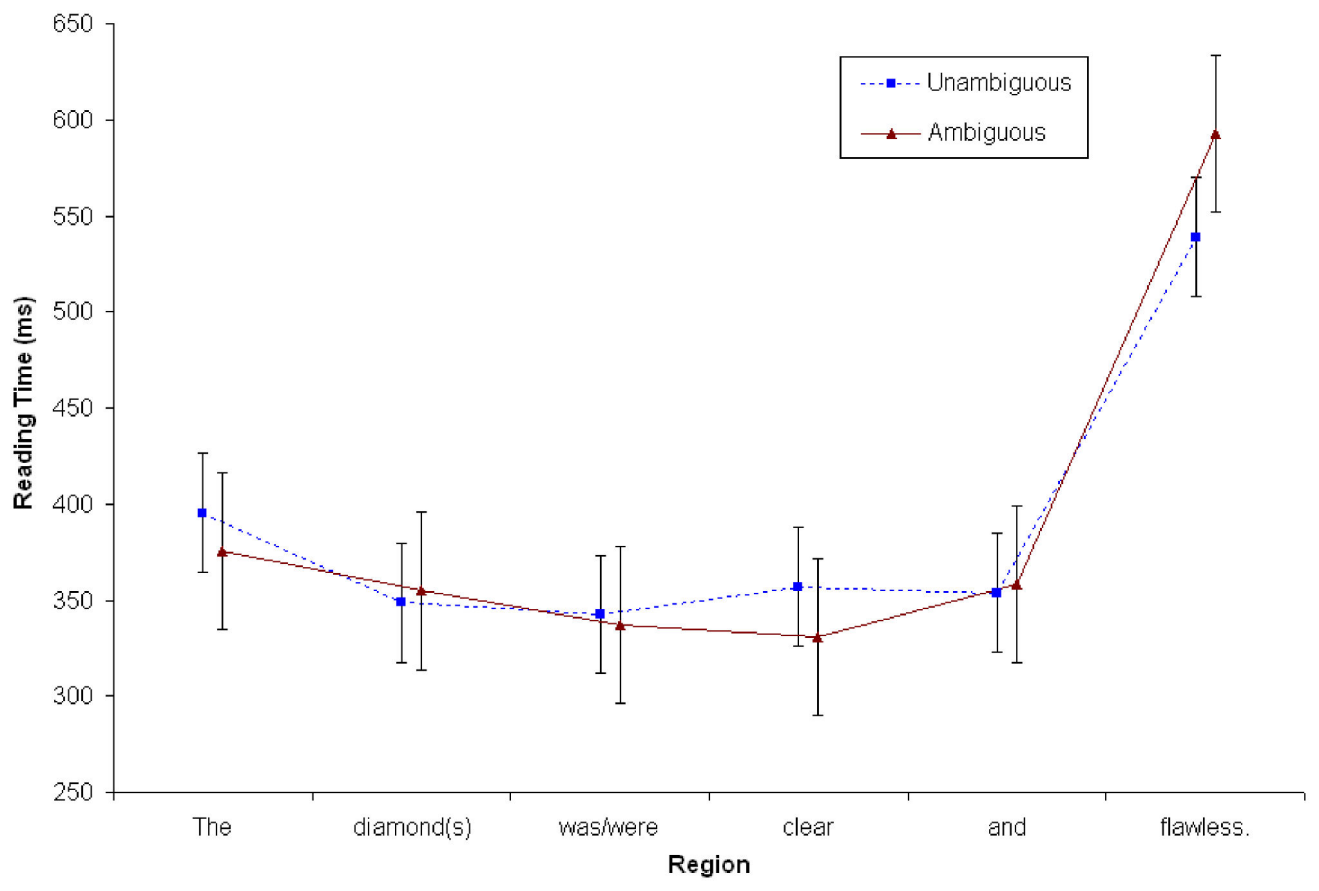
Reading time in milliseconds at S2 in Experiment 3 (*N*=40). Points represent the mean RTs per word; vertical lines depict standard error of the means.

A repeated measures ANOVA was conducted at the second sentence (S2), using the same criteria as in Experiments 1 and 2. There were no main effects associated with Context or Number (*Fs*<2). There was a significant interaction between Context x Word Position (*F*_*1*_ (5, 195) = 3.60, MSE = 1350; *p* = .011; ŋ_p_^2^=.085; *F*_*2*_ (5,115) = 4.81, MSE = 10624; *p* = .007; ŋ_p_^2^=.173). Simple effects analyses revealed that the final word (e.g., *flawless*) takes 50 ms longer to read in the ambiguous condition as compared to the unambiguous condition (579ms vs. 529ms). No other word positions showed any significant differences. Interestingly, there were no significant interactions with Number (*Fs*<2). See Figure 8.

#### Question answer results

**Figure 9:**
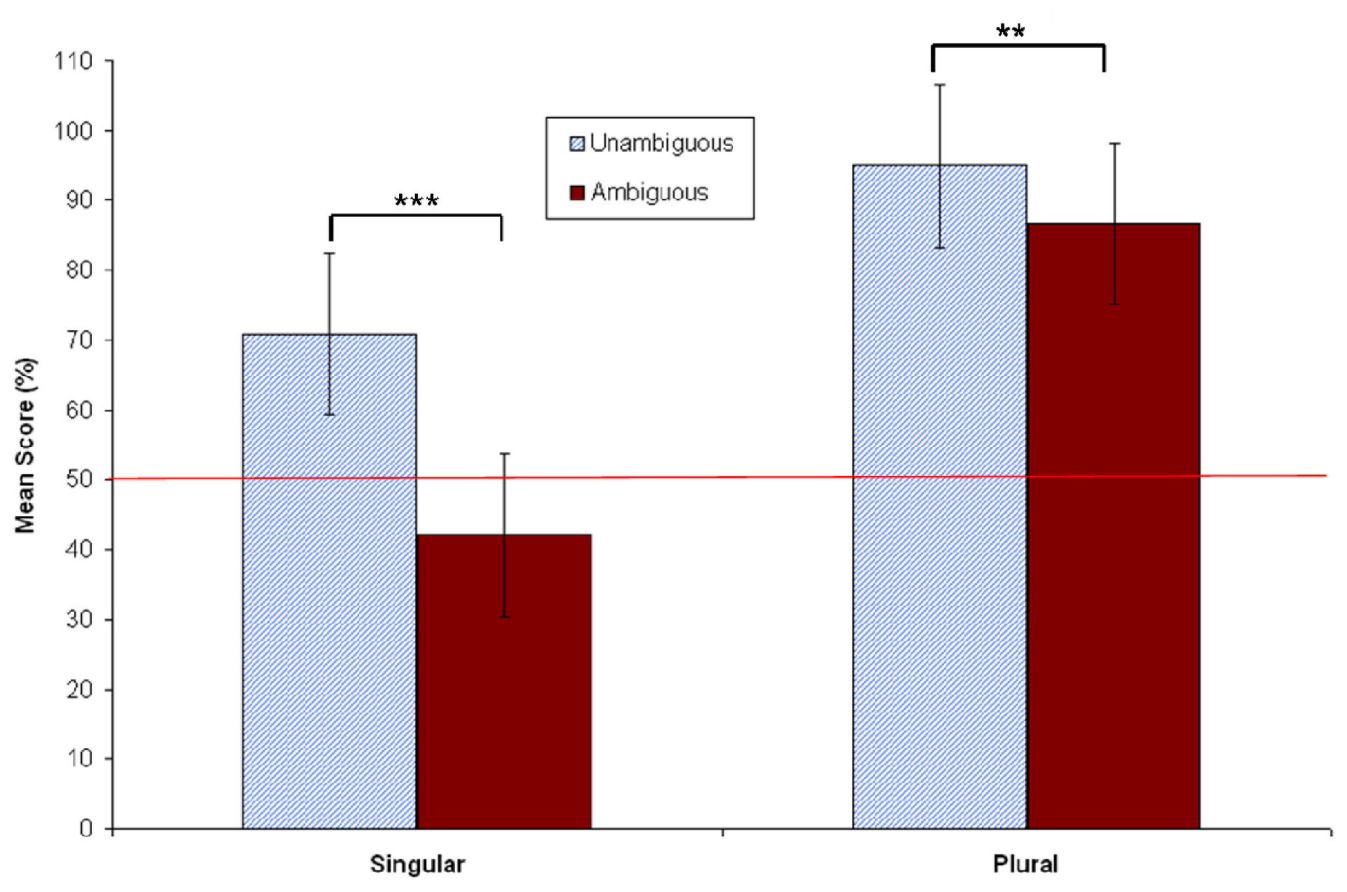
Mean comprehension question accuracy in Experiment 3 (*N*=40). Vertical lines depict standard error of the means, and the horizontal line indicates an accuracy score of 50% (chance). The difference between accuracy for unambiguous and ambiguous singular stimuli was significant at *p* < .001. The difference between accuracy for unambiguous and ambiguous plural stimuli was also significant at *p* < .01. **p < .01; ***p < .001.

**Figure 10:**
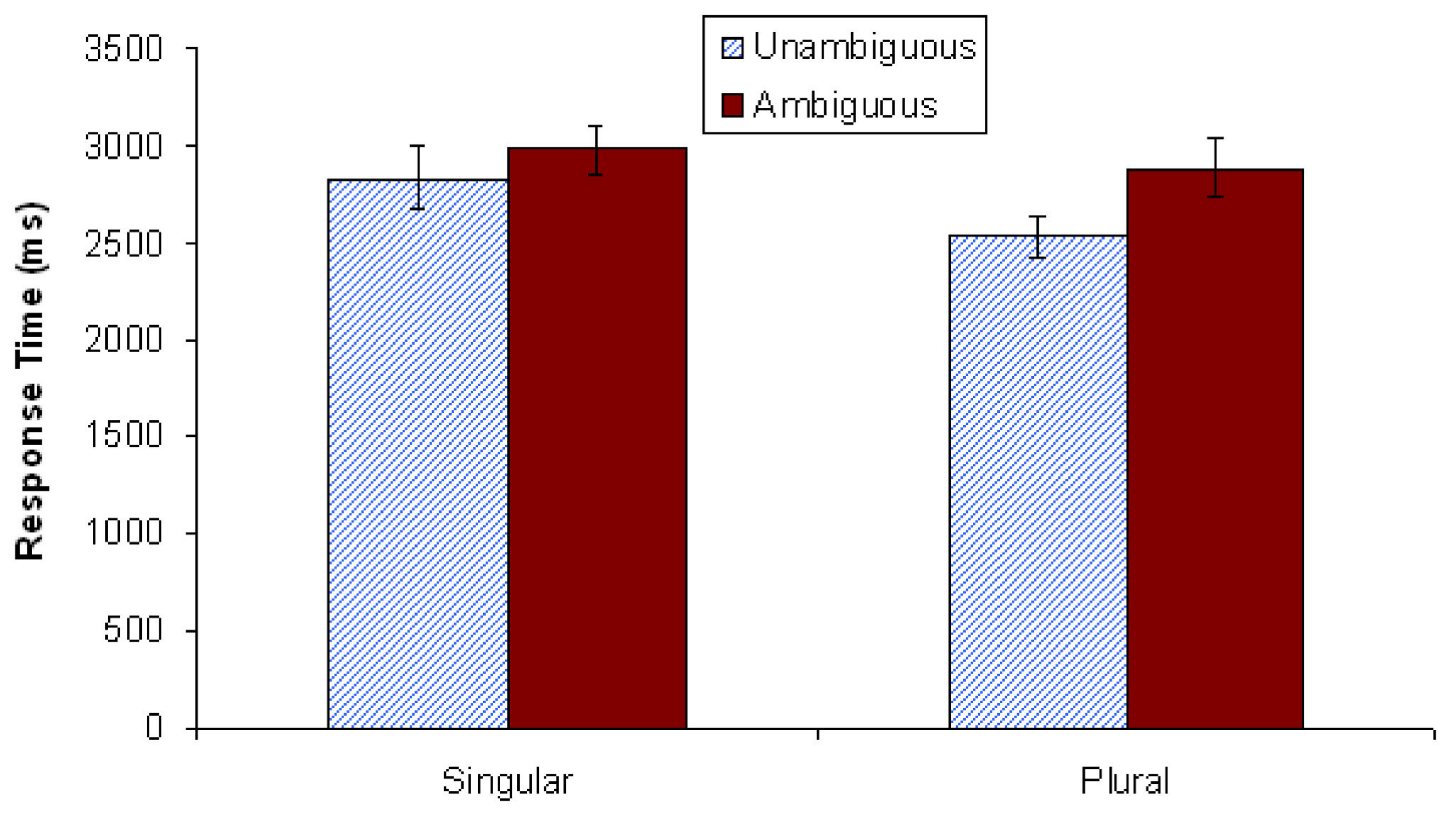
Mean comprehension question-response time in Experiment 3 (*N*=40). Vertical lines depict standard error of the means.

Figure 9 shows that participants performed at below chance levels again (down to 42%) when responding to the ambiguous singular condition. In contrast, the mean accuracy level for the ambiguous plural condition was 87% (see Text S12 in File S1). As Figure 10 shows, this difference in accuracy was not due to a speed-accuracy trade off, since singular conditions again took more time than plural conditions (see Text S13 in File S1). Both singular and plural question-response accuracy rates again differed significantly from their controls, as revealed in paired-samples t-tests (see Figure 9). The same bias towards accuracy in the plural conditions vs. the singular conditions was found; accuracy for the unambiguous singular condition was at 71% vs. 95% for the unambiguous plural (note that these values are almost exactly the same as those found in Experiment 2). 

### Discussion

To summarize the results above, in Experiment 3, results at S1 were similar to those of Experiments 1 and 2, where unambiguous context sentences took longer to read than ambiguous ones; in the present case, this effect was driven by the unambiguous singular condition. In addition, a reading time difference at S2 was found again, where it was consistent with the lexical-pragmatic bias of the stimuli. As such, ambiguous continuation sentences took longer to read than unambiguous sentences. Finally, despite the fact that the stimuli used in Experiment 3 were relatively unbiased, participants’ response accuracy rates were exactly the same as they were for Experiment 2: again, participants’ responses to ambiguous singular conditions occurred at below chance levels. The implication of these results is discussed in turn below.

#### Context sentence (S1)

Results at the first sentence mostly mirrored those found in Experiments 1 and 2 with a twist: the longer RTs for the unambiguous contexts seemed to be driven by the unambiguous singular condition, e.g., *Every jeweller appraised that diamond*. The longer RTs found for Experiments 1 and 2 at S1 were explained in the Discussion of Experiment 2—there is a discourse-pragmatic anomaly effect associated with the referential determiners *that* and *those*. In the present experiment, this effect is mainly driven by *that*. This result makes sense, given that participants do not have strong lexical-pragmatic biases with scenarios associated with *jeweller* ** *appraise* ** *diamond* (as opposed to *kid* ** *climb* ** *tree*). Filik, Sanford, and Leuthold [52] showed in an ERP language study that more effort is required to search and integrate a referential singular item into a discourse than a plural item, since plural reference is often non-specific. That is, singular pronouns (*s/he*) that did not have explicit antecedents produced larger N400 amplitudes than plural pronouns (*they*) without explicit antecedents. e.g., compare pronominal reference in *He served coffee at the event* vs. *They served coffee at the event*. Referential determiners perform the same linguistic and cognitive function in discourse as do pronouns (see Postal [53], who claimed that pronouns are in fact, determiners). As such, in the absence of any previous context *Every jeweller appraised that diamond* vs. *Every jeweller appraised those diamonds* results in longer reading times for the singular condition because *that diamond* picking out a singular item is pragmatically more odd in less well-defined scenarios from a discourse perspective. This is in contrast to the non-specific plural NP *those diamonds*. Thus, results at context sentences for Experiment 3 further bolster the explanation that the difference found between scope ambiguous vs. unambiguous context sentences in the present work is a difference that is due to discourse-pragmatic anomaly.

#### Continuation sentence (S2) and question-response accuracy

Regarding the effects at S2, a complexity effect was found where continuation sentences embedded in ambiguous sentences exhibited significantly longer RTs at the final word position as compared to those embedded in unambiguous contexts. In other words, like the number effect found in Experiment 2, results were consistent with the lexical-pragmatic biases of the context sentence. Thus, unlike Experiment 2, no difference was found for plural vs. singular continuation sentences. This makes sense if the RT data pattern for continuation sentences in Experiments 2 and 3 is in keeping with the (empirically observed) off-line biases of the context sentences— continuation sentences that are incongruent with lexical-pragmatic bias associated with the context sentence take longer to read. When the context is heavily biased for plural interpretation (Exp 2), singular conditions took longer to read. When context sentences are unbiased (Exp. 3), that is, they are truly ambiguous, then continuation sentences take longer to read when embedded in ambiguous vs. unambiguous contexts.

Results regarding question-answer response accuracy rates again did not pattern like those found for on-line reading times. That is, participants performed at below chance when answering questions to ambiguous singular conditions, and overall did much better answering questions following plural vs. singular conditions. Thus, accuracy rates again reflected biases associated with algorithmic parsing. This is in contrast to continuation sentence reading times, which, once more, reflected the lexical-pragmatic biases of the stimuli. The message to take away here is clear—even though the frequency of expectation regarding interpretation was quite different in Experiment 3 vs. Experiment 2, ultimate interpretation was exactly the same. This finding further underlines the importance of measuring participants’ actual interpretation of sentences, in addition to their reading times (see Ferreira [27] for similar claims).

## General Discussion

The present study examined the integration of sentences in discourses where context sentences exhibited quantifier scope ambiguity. The goal was to extend and clarify the claim that quantifier scope ambiguous sentences are processed in a shallow manner, and to use these underspecified constructions as a way of probing the relationship between heuristic and algorithmic processing strategies. 

Overall, results confirmed that quantifier scope ambiguous sentences are not deeply processed during interpretation. The processing of these sentences is so shallow that even when stimuli are controlled for, such that all are heavily biased for a surface scope interpretation, no effect of context was found at continuation sentences in Experiment 1. Nevertheless, this changed once questions were asked in Experiment 2. The pattern of results found in Experiment 2 was that RT data reflected the lexical-pragmatic bias of context, but question-response accuracy rates reflected algorithmic computation (such that ambiguous singular conditions were responded to at chance accuracy levels). In Experiment 3, RT data for continuation sentences again did not match the question-response accuracy rates. Furthermore, the question-response accuracy rates found in Experiment 3 were almost identical to those of Experiment 2. The lack of a difference between mean question-response accuracy rates between experiments was confirmed via an ANOVA, where no between group subject differences were found (*Fs*<1; see Text S14 in File S1). Furthermore, no between group subject differences regarding response times were revealed (Fs<1) either (see Text S15 in File S1). Thus, despite the difference in the lexical-pragmatic biases of the stimuli, the ultimate comprehension of the material was identical across experiments (see Text S16 in File S1). 

The fact that the reading times of continuation sentences reflected the lexical-pragmatic biases of context, whereas question-response accuracy did not, confirms two hypotheses put forth in the present article. First, quantifier scope ambiguous context sentences are processed using two independent streams of information processing: one that is a fast and frugal heuristic, sensitive to lexical-pragmatic biases of stimuli, and another that reflects deeper algorithmic processing, which is reflective of structural considerations. Second, the fast and frugal heuristic strategy precedes the application of algorithmic processing, should it occur at all.

The fast and frugal heuristic clarifies how quantifier scope ambiguous sentences are processed; these are interpreted according to lexical-pragmatic biases regarding number and (algorithmic) scope computation does not occur unless necessary. In other words, quantifier scope computation (see 1) is not a default strategy for sentences of the form *Every N*_*1*_ *Verbed an N*_*2*_, as that computation is an instance of algorithmic processing. Thus, processing scope ambiguous sentences is so shallow that integrating a continuation sentence with a definite NP anaphor *The tree*(*s*)*/The diamond(s*) is shallow too. This explains why no on-line RT differences were found for continuation sentences in Experiment 1.

On-line reading time differences were revealed in Experiments 2 and 3 because these experiments included specific questions regarding quantifier scope interpretation. As such, participants paid more attention to stimuli in these experiments. However, even when paying attention, heuristic processing prevailed during on-line reading, so that continuation sentences in these experiments that were incongruent with the lexical-pragmatic bias of the context sentences took longer to read. No effect of quantifier scope computation was revealed at continuation sentences. Instead, only when questions regarding scope interpretation were asked did participants finally do the work of computing quantifier scope. This algorithmic computation resulted in a pattern such that inverse scope (reflected in the ambiguous singular condition) was strongly dispreferred. Question-response accuracy rates for the ambiguous singular condition were below chance for both Experiments 2 and 3 (note that the relatively high accuracy rates for the other conditions indicate that these college-aged participants were indeed paying attention).

Thus, the findings confirm the claim made in Ferreira [27] that sentences are processed using both heuristic and algorithmic strategies. The present work goes beyond Ferreira [27] in the following way; first, the variety of the stimuli examined differs from that studied in other works on underspecification [36], [38], [54–57]. That is, quantifier scope ambiguous sentences differ markedly from relative clause constructions in that semantic, instead of syntactic, interpretation reliant on rules sensitive to grammatical structure is examined. This sort of algorithmic semantic interpretation is also called *compositional semantics* (see 7–9) as opposed to *conceptual semantics*, which is defined as meaning that is sensitive to the lexical-pragmatic biases derived from experience in the real-world. Secondly, this work addresses the issue of the temporal relationship between heuristic and algorithmic processing strategies. It examines how sentences are processed in discourse (vs. in isolation), so that lexical-pragmatic biases from context sentences are evident in reading time data for continuation sentences, and contrasted with algorithmic computation, associated with (the later-occurring) question-answer response data. 

The sequential timing of heuristic first, algorithmic later processing contrasts with proposals made in ERP language studies [58], [59], [28], [21], where it has been proposed that these independent processing streams (alternatively called *lexical-semantic* vs. *syntactic* processing streams) operate in parallel. As discussed earlier, the irony is that self-paced reading methodology revealed information regarding timing of processes where ERP methods could not. Again, this is because the standard slow rate of presentation of words (SOA of 600 ms) could allow for enough time for first pass heuristic strategies to apply (this could be in first 200-300 ms) followed by an algorithmic stage (especially for constructions that are not underspecified, see 6).

Thus, we can understand the findings of the ERP experiment investigating quantifier scope ambiguous discourses by Dwivedi et al. [11] in the following way: the fact that context sentences were read in their entirety, and under participants’ control (participants pressed a button once they were done reading the sentence) would allow for these sentences to be processed in a shallow manner. Then the slow presentation rate of the words in the continuation sentence enabled participants to process and integrate the second sentence, which required going back and paying attention to the meaning of the first sentence, which could entail computing quantifier scope. This difficult task would tax working memory resources, resulting in a slow negative shift which lasted throughout the presentation of the auxiliary verb *was/were*. The slow rate of presentation could explain why an effect of integration was elicited in the previous ERP language experiment but not in Experiment 1, presented here. Effects of integration were elicited in Experiments 2 and 3 in the present work due to the presence of questions, which forced more attention to be placed on the scope ambiguous discourse. Given that the effects found in the current self-paced studies were regarding the lexical-pragmatic properties of the context sentence, it could even be the case that the same was true of the ERP experiment. That is, it could be the case that the slow negative waveform was a marker of ambiguous vs. unambiguous contexts, instead of actual scope computation. Clearly, these findings open a host of new questions that can be asked regarding a model of processing semantic ambiguity. What is clear, for example, is that working memory and attention resources should have effects in terms of how such sentences are processed [14].

The effects of lexical-pragmatic biases underscore the importance of taking into account how number biases are present for certain events vs. others. That is, just like we have certain intuitions about plausibility regarding proto-typical subjects vs. objects (where for example, the former is usually animate and the latter inanimate) we have intuitions about the number of participants in events, too. Recognizing the presence of lexical-pragmatic biases with respect to number for events is useful because number is employed as a way of disambiguating quantifier scope ambiguous sentences in language processing experiments. If this lexical-pragmatic contribution is not taken into account, it is unclear what one is measuring, (see also recent work by Patson & Warren [60], which examined the interpretation of collective vs. distributive verbs in quantificational sentences).

It is noteworthy that the present work, unlike previous studies, did not attempt to decide amongst theories of quantifier scope processing such as the *thematic role account* vs. the *linear order account* or *quantifier hierarchy account* [61], [13], [10], [15], [12], because the assumption here is that quantifier scope ambiguity as a processing phenomenon is not well-understood enough to decide amongst such theoretical proposals. Therefore, other basic properties regarding quantifier scope interpretation need to be better understood first, before these theoretical proposals can be addressed. For example, besides controlling for number biases associated with particular events, another important factor to investigate is the semantic interpretation associated with the quantifiers in question, namely *a* and *every*. For instance, *a N* can have a specific (i.e., referential and thus, non-quantificational) vs. non-specific (i.e., quantificational) interpretation [2]. As such, if the quantifier order is reversed, as in *A kid climbed every tree*, having *a kid* in topic/subject position would bias the interpretation of this noun phrase as referential, and not quantificational. On a non-quantificational interpretation, there would be no scope ambiguity. Nevertheless, the number associated with *a kid* would still be singular. In other words, the number associated with *a kid* on the non-quantificational interpretation, as well as the so-called surface scope reading would be identical. As such, if participants choose an interpretation with one kid and lots of trees, it is not clear whether the interpretation arises from a pragmatic interpretation of reference or algorithmic computation of scope. In the present work (see Tables 1 and 2, as well as Critical Stimuli Lists in File S1) the indefinite NP *a N* was always in direct object position and inanimate because these factors would bias for a quantificational reading of the determiner *a*. In any case, until this is clarified, it is unclear what to make of studies that examine sentences with the configuration *A N*_*1*_ *Verbed every N*_*2*_ and claim a certain preference for interpretation (see literature cited above). It has been shown that interpretation regarding number has a variety of linguistic sources; as such, these sources need to be teased apart in order to better understand quantifier scope interpretation. 

Furthermore, the semantic properties of *every* also need to be understood with respect to processing. Filik et al. [13] and Paterson et al. [15] made claims regarding a different syntactic configuration (now quantifier order is varied in two different kinds of VPs), however, a close examination of their stimuli reveals that the semantic requirements of *every* were not met across conditions. Without going into the details of their proposal, the important thing to note for this eye-movement study is that stimuli that took longer to process were configurations where *every* did not have a restrictive term. For example, Filik et al. [13] found that the (underlined) region containing the two quantifiers (regions are delineated by | marks) took longer to read for (5b) vs. (5a):

(5) a. The celebrity gave| an indepth interview to every reporter from the newspaper but | the interview(s) was/were | not very | interesting.
b. The celebrity gave | every indepth interview to a reporter from the newspaper but| the reporter(s) was/were | not very | interested.

Again, it is unclear whether this finding is actually related to quantifier scope computation. Since *every* is a strong quantificational determiner, it requires a restrictive term, or a context/domain over which to quantify [62–64]. Example (5a) provides a PP *from the newspaper* which can serve as the domain of *every*, but (5b) does not. Thus, increased RTs for (5b) could reflect the pragmatic anomaly for *every* and have nothing to do with differences in scope computation (see Text S17 in File S1). Thus, along with the number biases associated with events, the semantic properties of the quantifiers that are under investigation need to be taken into account.

In another recent study, Raffray and Pickering [12] claimed that quantifier scope computation at the level of Logical Form (LF) could be primed. They argued that given that earlier work indicated that syntactic analysis could be primed by exposure to a previous structure (see 65], [66), similar effects should prevail for structure built at LF. In a picture priming task, they found that comprehenders’ final interpretation of target sentences as in *Every hiker climbed a hill* (note that target sentences always had the form *Every N*_*1*_ *Verbed an N*_*2*_ ) could be primed by previous pictures denoting a surface scope or inverse scope interpretation of quantifier scope ambiguous sentences. That is, when a picture depicting singular number for *tree* in *Every kid climbed a tree* was shown right before the target *Every hiker climbed a hill*, the choice for a plural interpretation for *tree* would drop from 77% to 69%.

In the present study it has been argued that there are two routes to understanding scope ambiguous sentences. Thus, whereas Raffray and Pickering [12] proposed that algorithmic computation can be primed (an argument which seems plausible) it is entirely possible that effects elicited were due to a lexical-pragmatic bias for number associated with the sentences and not due to algorithmic computation. This alternative hypothesis is especially relevant, given that for all 4 experiments reported, only 12 verbs were used for 24 items in each experiment, where the prime and the target always had the same verb. Participants chose the plural interpretation for target sentences across experiments the majority of the time. Although Raffray and Pickering [12] never asked why the plural was so strongly preferred across experiments, it could be the case that preferences reflected the lexical-pragmatic biases associated with the 12 verbs that they used. Thus, any drop in frequency of plural interpretation could reflect a drop in the bias for the lexical-pragmatic number associated with the target sentence, rather than the effect of an algorithmic computation of that sentence. In addition, although participants were asked about their interpretation of sentences, the interpretation was presented visually in terms of a picture (event) scenario. It is not clear that algorithmic computation would be required for this visual task. Finally, results from their final experiment strongly call into question the conclusion that any LF algorithmic computation took place at all. For that experiment, the prime sentence had plural number overtly marked on *N*_*1*_ and *N*_*2*_, as in *Kids like to climb trees* (i.e., no scope ambiguity, no quantifiers were present in prime sentences). Now, choices for the target *Every hiker climbed a hill* for plural hikers/plural hills increased to 84%. The fact that sentences *without* a similar syntactic and semantic structure more strongly engendered a plural interpretation as compared to ones with *exactly the same structure* seriously undermines the claim that priming occurred due to algorithmic computation. Instead, it seems that when the lexical-pragmatic bias for number is overt, that is, where both Ns are marked as plural, the interpretation for sentences of the form *Every N*_*1*_ *Verbed an N*_*2*_ gets a boost in terms of the plural interpretation associated with *N*_*2*_. Thus, it seems that the proposal presented in this paper accounts for the data presented in Raffray and Pickering [12]. 

## Conclusion

In sum, the role of heuristic vs. algorithmic processing mechanisms was examined with respect to the processing of quantifier scope ambiguous sentences. These mechanisms are efficiently co-ordinated such that heuristic processing of these sentences occurs first and algorithmic ‘deep’ computation of quantifier scope does not occur in real-time unless it is required. It was proposed that when examining quantifier scope ambiguous sentences, the lexical-pragmatic bias of number with respect to event scenarios cannot be ignored. This bias can result in a shallow interpretation of quantifier scope ambiguous sentences with respect to number interpretation. Deeper processing, reflecting algorithmic computation, occurs only if necessary. Not only does this model account for results of the three experiments discussed in this study, it adequately accounts for other recent findings in the literature examining quantifier scope ambiguity processing. Furthermore, it opens up a host of new questions; for example, how do these mechanisms interact with sentence picture verification (see Dwivedi, Endicott, Curtiss & Gibson, [67], as well as Dwivedi & Curtiss, [68], which follow up on this issue). 

Thus, it has been proposed that language comprehension consists of two independent routes—one fast and frugal heuristic and another slower algorithmic computation. As such, it appears that the architecture associated with language processing is comparable to that argued for other cognitive processes. Furthermore, the importance of timing and experience in language processing was discussed. Given that language processing is ultimately a human brain function, the significance of these factors is expected, as Kutas [69] has argued that these factors are important in describing brain processes in general. 

## Supporting Information

File S1 **Supporting information**.
File S1 contains notes from Text S1-Text S17, as well as Critical Stimuli List S1 and Critical Stimuli List S2.
(DOCX)
