# Supplementary material for: Interpreting Quantifier Scope Ambiguity: Evidence of Heuristic First, Algorithmic Second Processing
Source: PLoS One. 2013 Nov 20;8(11):e81461. doi: 10.1371/journal.pone.0081461 (PMC3835328; doi:10.1371/journal.pone.0081461)
Supplement: File S1 — Supporting information. File S1 contains notes from Text S1-Text S17, as well as Critical Stimuli List S1 and Critical Stimuli List S2. (DOCX) [file pone.0081461.s001.docx]

**TITLE:** Interpreting Quantifier Scope Ambiguity: Evidence of Heuristic First, Algorithmic Second Processing

Supporting Information

**AUTHOR:** Veena D. Dwivedi, PhD

Department of Applied Linguistics and the Centre for Neuroscience

Brock University, Niagara Region, 500 Glenridge Ave., St. Catharines, Ontario, Canada,

L2S 3A1

Phone: (905)688-5550, ext. 5389

Email: veena.dwivedi@brocku.ca**Text S1**

This is expected, given the shorter movement of the quantifier from subject position to an adjoined S position. Regardless of linguistic theory however, given that the surface scope interpretation is consistent with the surface linear order of the sentence, by Occam’s razor, this should be the preferred interpretation. This would always be the simplest representation for interpretive purposes.

**Text S2**

Note that some linguists and logicians argue that *Every kid climbed a tree* is not necessarily disambiguated on its surface scope reading by the plural continuation *The trees were in the park* because the singular continuation could refer to a discourse subordinate reading for *tree* where the discourse would read as *Every kid climbed a tree, (and) The (particular) tree (that they all climbed) was in the park.* While this reading might be available on theoretical and intuitive grounds, the empirical facts indicate otherwise. First, there are the biases reported in Kurtzman and MacDonald [1], as well as the normative data provided in Dwivedi et al. [2]. Second, if this interpretation were in fact available, then this should have been observable in the on-line ERP data discussed above. In fact, a similar ‘discourse subordinate’ claim was made in Dwivedi et al. which examined discourses exhibiting modal subordination (arguably another type of quantifier scope ambiguity, except at the level of possible worlds instead of entities). The ‘dispreferred’ continuation in that study exhibited a P600 effect, which was not replicated in Dwivedi et al.[2]. In short, while the point is well taken on purely intuitive and theoretical grounds, there is to date, no empirical evidence to support this claim.

1. Kurtzman HS, MacDonald MC (1993) Resolution of quantifier scope ambiguities. Cognition 48: 243-279. 10.1016/0010-0277(93)90042-T.

2. Dwivedi VD, Phillips NA, Einagel S, Baum SR (2010) The neural underpinnings of semantic ambiguity and anaphora. Brain Research 1311: 93-109. 10.1016/j.brainres.2009.09.102.

**Text S3**

The notation shown in (1) can be represented any number of ways. It is not the goal of this paper to take a stand on this.

**Text S4**

See the work of Szymanik [3] where a very similar proposal is made in terms of a computational cognitive model.

3. Szymanic J (2010) Computational complexity of polyadic lifts of generalized quantifiers in natural language. Linguistics and Philosophy 33: 215-250. 10.1007/s10988-010-9076-z.

**Text S5**

Note that the methodological reason for this was that Kaan and Swaab [4] showed a neurophysiological artifact (a Left Anterior Negativity effect) for critical trials that were followed by questions. As such, this was avoided in Dwivedi et al. [2].

2. Dwivedi VD, Phillips NA, Einagel S, Baum SR (2010) The neural underpinnings of semantic ambiguity and anaphora. Brain Research 1311: 93-109. 10.1016/j.brainres.2009.09.102.

4. Kaan E, Swaab TY (2003) Electrophysiological Evidence for Serial Sentence Processing: A Comparison Between Non-Preferred and Ungrammatical Continuations. Cognitive Brain Research 17: 621-635. 10.1016/S0926-6410(03)00175-7.

**Text S6**

Note that for Experiments 1 and 2, where heuristic biases favour the plural interpretation, predictions for ‘syntax first’ models are the same, since the algorithmic computation also favours the plural. The contrast of interest in the present paper is the parallel view vs. heuristic first, algorithmic second and only if required.

**Text S7**

This was necessary because in the previous ERP study, the unambiguous singular control condition (modeled on stimuli found in Kurtzman & MacDonald [1]) patterned with the two ambiguous conditions, see Dwivedi et al. [2] for details. It was argued that *the same N* could have a quantificational reading. Thus, in order to improve upon the unambiguous stimuli, referential (i.e., non-quantificational) *that* and *those* are used here.

1. Kurtzman HS, MacDonald MC (1993) Resolution of quantifier scope ambiguities. Cognition 48: 243-279. 10.1016/0010-0277(93)90042-T.

2. Dwivedi VD, Phillips NA, Einagel S, Baum SR (2010) The neural underpinnings of semantic ambiguity and anaphora. Brain Research 1311: 93-109. 10.1016/j.brainres.2009.09.102.

**Text S8**

A brief working memory task was administered prior to the self-paced RT task; derived from Daneman and Carpenter ([5], [6]). The task was employed to ask questions orthogonal to the present paper and so results are not reported with respect to working memory.

5. Daneman M, Carpenter PA (1980) Individual Differences in Working Memory and Reading. Journal of Verbal Learning and Verbal Behavior 450-466. 10.1016/S0022-5371(80)90312-6.

6. Siegel LS, Ryan EB (1989) The Development of Working Memory in Normally Achieving and Subtypes of Learning Disabled Children. Child Development 60: 973-980.

**Text S9**

PASW is formerly known as SPSS.

**Text S10**

In addition to the previously mentioned working memory task, another on-line working memory task was administered (where order of presentation of the task was counterbalanced across participants). Again, results are not reported as they are orthogonal to the question asked in the present paper.

**Text S11**

An ANOVA for question response times for Exp 2 the results revealed a main effect of Context, *F_1_* (1, 47) = 12.10, *MSE* = 490586; *p* < .001; ŋ_p_^2^=.205, as well as a main effect of Number *F_1_* (1, 47) = 5.75, *MSE* = 862281; *p* =.021; ŋ_p_^2^=.109. There were no interactions (F<1). Effects were exactly parallel by item. The main effect of Context is consistent with the accuracy results, which showed that ambiguous conditions were less accurate. These also took more time to respond to. Similarly, the main effect of Number is also consistent with the accuracy results, since singular conditions took more time to respond to.

**Text S12**

A reviewer raises the interesting question as to why accuracy rates for the ambiguous plural condition, while high, are not at ceiling as they are for the unambiguous control plural condition. The difference in accuracy rates between the two conditions would be explained as a cost associated with perceiving sentential ambiguity. This cost is noted in the extra time required to respond to questions following ambiguous contexts (see results above). Further evidence is provided via ERP language work by Frisch et al.[7], Bornkessel et al.[8], as well as Dwivedi et al.[2], who have shown that differing waveforms result when processing ambiguous vs. unambiguous sentences. For our purposes, one way of understanding the results would be that since more capacity is required to compute the meaning of ambiguous vs. unambiguous sentences (where this computation is in response to the question), this would result in fewer resources available to attend to the task [9] —resulting in high but not ceiling accuracy rates.

2. Dwivedi VD, Phillips NA, Einagel S, Baum SR (2010) The neural underpinnings of semantic ambiguity and anaphora. Brain Research 1311: 93-109. 10.1016/j.brainres.2009.09.102.

7. Frisch S, Schlesewsky M, Saddy D, Alpermann A (2002) The P600 as an indicator of syntactic ambiguity. Cognition 85: B83-B92.

8. Bornkessel ID, Fiebach CJ, Friederici AD (2004) On the cost of syntactic ambiguity in human language comprehension: an individual differences approach. Cognitive Brain Research 21: 11-21.

9. Sternberg S (1969) The discovery of processing stages: Extensions of Donders' method. Acta Psychologica 30: 276-315.

**Text S13**

An ANOVA for question response times for Exp 3 results revealed a main effect of Context, *F_1_* *F* (1, 39) = 8.07, *MSE* = 309266; *p* =.007; ŋ_p_^2^=.171, as well as a main effect of Number *F* (1, 39) = 4.62, *MSE* = 362991; *p* =.038; ŋ_p_^2^=.106. There were no interactions (*F*<2). Effects were exactly parallel by item. See discussion in Text S11 above.

**Text S14**

Context x Number x Experiment was not significant (*F*(1, 86) = 0.8; *MSE* = 0.03; *p* = 0.38; *ŋ_p_^2^* = 0.009). There were no other interactions with the factor Exp, all *Fs*<2.

**Text S15**

Context x Number x Experiment was not significant (F(1, 86) = 0.08; MSE = 472 628; *p* = 0.8; ŋ_p_^2^ = 0.001). There were no other interactions with Exp, all Fs<1.

**Text S16**

This in itself is interesting, as it speaks to the issue of whether frequency of expectation affects reading comprehension. In the present case, where the structure was identical but lexical-pragmatic biases differed, clearly there was no effect of the ease of predictability in interpretation of the heavily biased stimuli vs. the less biased stimuli. The less biased stimuli should have been more difficult to process, according to Gennari and MacDonald [10]. This is because on their account, the less biased stimuli should have activated both plural and singular continuations, whereas the heavily biased stimuli would have only activated the plural interpretation.

10. Gennari SP, MacDonald MC (2008) Semantic indeterminacy in object relative clauses. Journal of Memory and Language 58: 161-187. 10.1016/j.jml.2007.07.004.

**Text S17**

Note that the lack of a restrictive term for *every* was also true for the double object condition under investigation, not discussed here for reasons of brevity. Furthermore, Filik et al.[11] found that despite their claim for scope computation in the second region, the region containing the definite NP anaphor *the interview(s) was/were* only showed a preference for the singular condition. Under an explanation where scope is not computed, this makes sense since co-reference was always to a singular antecedent (see 5a,b)—in which case, a singular anaphor would be preferred. As a side note, another problem with the above mentioned studies was the lack of an unambiguous control condition. In other words, ambiguous sentences were always compared to other ambiguous sentences (albeit configured differently).

11. Filik R, Paterson KB, Liversedge SP (2004) Processing doubly quantified sentences: Evidence from eye movements. Psychonomic Bulletin and Review 11: 953-959. 10.3758/BF03196727.

**Critical Stimuli List S1**

Critical stimuli for Experiments 1 and 2 are shown. Context sentences appear first, followed by continuation sentences. Percentages in parentheses indicate normed preferences for plural interpretation from the norming study reported in Dwivedi et al. [2]. Questions were only presented in Experiment 2.

1. Every delinquent scratched a/that/those car(s). The car(s) was/were new and expensive. (100%)

How many cars were scratched? One Several

1. Every maid mopped a/that/those hallway(s). The hallway(s) was/were long and narrow. (100%)

How many hallways were mopped? One Several

1. Every carpenter built a/that/those barn(s). The barn(s) was/were red and white. (100%)

How many barns were built? One Several

1. Every supervisor disciplined a/that/those worker(s). The worker(s) was/were lazy and careless. (100%)

How many workers were disciplined? One Several

1. Every customer returned an/that/those item(s). The item(s) was/were faulty or broken. (100%)

How many items were returned? One Several

1. Every tailor altered a/that/those jacket(s). The jacket(s) was/were feminine and sophisticated. (100%)

How many jackets were altered? One Several

1. Every stylist chose a/that/those shirt(s). The shirt(s) was/were crisp and clean. (100%)

How many shirts were chosen? One Several

1. Every camper carried a/that/those canoe(s). The canoe(s) was/were green and shiny. (100%)

How many canoes were carried? One Several

1. Every kid climbed a/that/those tree(s). The tree(s) was/were in a playground. (100%)

How many trees were climbed? One Several

1. Every landscaper trimmed a/that/those hedge(s). The hedge(s) was/were in a garden. (100%)

How many hedges were trimmed? One Several

1. Every entrepreneur opened a/that/those store(s). The store(s) was/were in a mall. (100%)

How many stores were opened? One Several

1. Every man photographed a/that/those monument(s). The monument(s) was/were large and spectacular. (94%)

How many monuments were photographed? One Several

1. Every spy received an/that/those assignment(s). The assignment(s) was/were hazardous and risky. (94%)

How many assignments were received? One Several

1. Every citizen endured a/that/those hurricane(s). The hurricane(s) was/were destructive and devastating. (94%)

How many hurricanes were endured? One Several

1. Every executive attended a/that/those conference(s). The conference(s) was/were about new products. (94%)

How many conferences were attended? One Several

1. Every rider jumped a/that/those fence(s). The fence(s) was/were beside a ranch. (94%)

How many fences were jumped? One Several

1. Every pedestrian exited a/that/those tunnel(s). The tunnel(s) was/were underneath hard rock. (94%)

How many tunnels were exited? One Several

1. Every athlete recited a/that/those anthem(s). The anthem(s) was/were special and moving. (93%)

How many anthems were recited? One Several

1. Every stockbroker advised a/that/those client(s). The client(s) was/were rich and famous. (93%)

How many clients were advised? One Several

1. Every grandmother baked a/that/those dessert(s). The dessert(s) was/were sweet and delicious. (93%)

How many desserts were baked? One Several

1. Every competitor thanked a/that/those sponsor(s). The sponsor(s) was/were generous and supportive. (93%)

How many sponsors were thanked? One Several

1. Every astronomer found a/that/those planet(s). The planet(s) was/were far and gaseous. (93%)

How many planets were found? One Several

1. Every warrior defended a/that/those kingdom(s). The kingdom(s) was/were vast and expansive. (93%)

How many kingdoms were defended? One Several

1. Every comedian did an/that/those impression(s). The impression(s) was/were perfect and hilarious. (93%)

How many impressions were done? One Several

**Critical Stimuli List S2**

Critical stimuli for Experiment 3 are shown. Context sentences appear first, followed by continuation sentences. Percentages in parentheses indicate normed preferences for plural interpretation from the norming study reported in Dwivedi et al. [2].

1. Every hunter fired a/that/those gun(s). The gun(s) was/were loud and startling. (67%)

How many guns were fired? One Several

1. Every jeweller appraised a/that/those diamond(s). The diamond(s) was/were clear and flawless. (67%)

How many diamonds were appraised? One Several

1. Every janitor washed a/that/those floor(s). The floor(s) was/were old and worn. (67%)

How many floors were washed? One Several

1. Every individual celebrated a/that/those holiday(s). The holiday(s) was/were happy and festive. (67%)

How many holidays were celebrated? One Several

1. Every witch cast a/that/those spell(s). The spell(s) was/were spooky and mysterious. (67%)

How many spells were cast? One Several

1. Every thief grabbed a/that/those sack(s). The sack(s) was/were beside a safe. (67%)

How many sacks were grabbed? One Several

1. Every ranger cleared a/that/those trail(s). The trail(s) was/were through a valley. (67%)

How many trails were cleared? One Several

1. Every singer gave a/that/those concert(s). The concert(s) was/were for disaster relief. (67%)

How many concerts were given? One Several

1. Every researcher published a/that/those journal(s). The journal(s) was/were about scientific discoveries. (67%)

How many journals were published? One Several

1. Every president ignored a/that/those warning(s). The warning(s) was/were about national security. (67%)

How many warnings were ignored? One Several

1. Every girl hummed a/that/those melody(s). The melody(s) was/were soft and sweet. (61%)

How many melodies were hummed? One Several

1. Every musician played a/that/those song(s). The song(s) was/were honest and heartfelt. (61%)

How many songs were played? One Several

1. Every resident survived a/that/those storm(s). The storm(s) was/were powerful and destructive. (61%)

How many storms were survived? One Several

1. Every friend grieved a/that/those death(s). The death(s) was/were sudden and unexpected. (61%)

How many deaths were grieved? One Several

1. Every king hosted a/that/those banquet(s). The banquet(s) was/were loud and boisterous. (61%)

How many banquets were hosted? One Several

1. Every detective examined a/that/those clue(s). The clue(s) was/were in a diary. (61%)

How many clues were examined? One Several

1. Every employee completed a/that/those project(s). The project(s) was/were for a client. (61%)

How many projects were completed? One Several

1. Every scientist asked a/that/those question(s). The question(s) was/were about a theory. (61%)

How many questions were asked? One Several

1. Every relative took a/that/those vacation(s). The vacation(s) was/were during the summer. (61%)

How many vacations were taken? One Several

1. Every neighbour lent a/that/those tool(s). The tool(s) was/were sharp and shiny. (56%)

How many tools were lent? One Several

1. Every speaker quoted a/that/those phrase(s). The phrase(s) was/were bland and overused. (56%)

How many phrases were quoted? One Several

1. Every soldier saluted a/that/those flag(s). The flag(s) was/were above a fort. (50%)

How many flags were saluted? One Several

1. Every student borrowed a/that/those book(s). The book(s) was/were about war heroes. (50%)

How many books were borrowed? One Several

1. Every chef avoided a/that/those recipe(s). The recipe(s) was/were bland and unappetizing. (44%)

How many recipes were avoided? One Several

Reference List

1. Kurtzman HS, MacDonald MC (1993) Resolution of quantifier scope ambiguities. Cognition 48: 243-279. 10.1016/0010-0277(93)90042-T.

2. Dwivedi VD, Phillips NA, Einagel S, Baum SR (2010) The neural underpinnings of semantic ambiguity and anaphora. Brain Research 1311: 93-109. 10.1016/j.brainres.2009.09.102.

3. Szymanik J (2010) Computational complexity of polyadic lifts of generalized quantifiers in natural language. Linguistics and Philosophy 33: 215-250. 10.1007/s10988-010-9076-z.

4. Kaan E, Swaab TY (2003) Electrophysiological Evidence for Serial Sentence Processing: A Comparison Between Non-Preferred and Ungrammatical Continuations. Cognitive Brain Research 17: 621-635. 10.1016/S0926-6410(03)00175-7.

5. Daneman M, Carpenter PA (1980) Individual Differences in Working Memory and Reading. Journal of Verbal Learning and Verbal Behavior 450-466. 10.1016/S0022-5371(80)90312-6.

6. Siegel LS, Ryan EB (1989) The Development of Working Memory in Normally Achieving and Subtypes of Learning Disabled Children. Child Development 60: 973-980.

7. Frisch S, Schlesewsky M, Saddy D, Alpermann A (2002) The P600 as an indicator of syntactic ambiguity. Cognition 85: B83-B92.

8. Bornkessel ID, Fiebach CJ, Friederici AD (2004) On the cost of syntactic ambiguity in human language comprehension: an individual differences approach. Cognitive Brain Research 21: 11-21.

9. Sternberg S (1969) The discovery of processing stages: Extensions of Donders' method. Acta Psychologica 30: 276-315.

10. Gennari SP, MacDonald MC (2008) Semantic indeterminacy in object relative clauses. Journal of Memory and Language 58: 161-187. 10.1016/j.jml.2007.07.004.

11. Filik R, Paterson KB, Liversedge SP (2004) Processing doubly quantified sentences: Evidence from eye movements. Psychonomic Bulletin and Review 11: 953-959. 10.3758/BF03196727.
